# Inoculation with old‐growth microbes improves restoration quality and reduces non‐native encroachment after seven years

**DOI:** 10.1002/eap.70295

**Published:** 2026-09-08

**Authors:** Tanya E. Cheeke, Reb L. Bryant, Gunner Davies, Liz Koziol, James D. Bever

**Affiliations:** ^1^ School of Biological Sciences, Washington State University Richland Washington USA; ^2^ Department of Ecology and Evolutionary Biology University of Kansas Lawrence Kansas USA; ^3^ Present address: Confederated Salish and Kootenai Tribes Polson Montana USA

**Keywords:** grassland, inoculation, microbe, mycorrhizal fungi, prairie restoration, site preparation

## Abstract

Understanding the mechanisms that promote native seedling establishment in disturbed ecosystems is key to improving the biodiversity and function of landscape restorations. Over seven growing seasons, we examined the effects of site preparation technique, soil microbial inoculations, and their interactions on native plant establishment in the restoration of an invaded grassland. Prior to restoration, field plots were either (a) solarized to clear the non‐native grass *Bromus inermis* or (b) mowed, and one of three soil microbial treatments was introduced into each plot using native tallgrass prairie seedlings as “nurse” plants, inoculated with (1) arbuscular mycorrhizal (AM) fungi isolated from old‐growth prairie remnants, (2) remnant prairie soil, or (3) autoclaved inocula. Uninoculated phytometer seedlings were planted at different distances from the nurse plant row to test for plant–soil feedbacks and spread of microbes into the restoration site. A diverse native seed mix was applied to test whether site preparation technique and/or microbial inoculations impacted the composition of the plant community. We found that site preparation technique and microbial inoculation were strong drivers of plant community composition, and saw evidence of plant–soil feedbacks in the first year of the study. By the seventh growing season, there was greater cover of late successional native plant species and increased floristic quality index scores in solarized plots and inoculated plots compared to mowed and uninoculated plots. Overseeding without solarization was mostly effective for establishing native grasses, which comprised 66% of plot abundance after three overseeding attempts. By considering belowground communities at the same time as revegetation efforts, this study demonstrates how soil microbial inoculations can be used in conjunction with site preparation technique as a nature‐based tool to accelerate the recovery of invaded grasslands.

## INTRODUCTION

Ecological disturbance, such as conversion of prairies to agricultural land, is a major cause of habitat loss, and the grasslands of North America are one of the most imperiled ecosystems on Earth (Carbutt et al., 2017). Historically, tallgrass prairies dominated the landscape in the Midwestern USA, but since the 1940s, 40 million acres of native prairies has been replaced by near monocrops of non‐native grasses such as *Bromus inermis* (smooth brome), leading to a loss of biodiversity (McCain et al., 2011), reduction of multitrophic interactions (Cardinale et al., 2006), and disruption of ecosystem services (Balvanera et al., 2006; Hooper et al., 2005). Native to Europe, *B. inermis* is a perennial cool season C3 grass that was promoted as a forage crop in the early 20th century because of its ease of cultivation, hardiness, and drought‐tolerance (Mackiewicz‐Walec et al., 2024). However, it has since become a pervasive weed in grasslands throughout the Midwestern United States (Palit & DeKeyser, 2022). Although efforts to restore native prairies are increasing, non‐native plant species are often aggressive competitors that are difficult to remove or control (Mack et al., 2000). Some non‐native plants also have life history traits or competitive strategies, such as creating dense sod or altering soil microbial communities that promote their own dominance in a landscape (van der Putten et al., 2013).

One way to improve the establishment of native plants in invaded or disturbed ecosystems may be to inoculate them with beneficial soil organisms, such as native arbuscular mycorrhizal (AM) fungi, prior to planting. AM fungi associate with more than 80% of terrestrial plants and play a fundamental role in plant nutrient uptake (Smith & Read, 2008), contribute to soil structure, fertility, and carbon storage (Johnson et al., 2017), and improve resilience to environmental stress (Delavaux et al., 2017). However, biological invasions and agricultural practices such as tillage and fertilizer applications can negatively impact the abundance and community composition of AM fungi and other soil organisms (Berner et al., 2025; House & Bever, 2018; Oehl et al., 2005; Verbruggen & Kiers, 2010). This can hinder restoration efforts because some native plants are highly dependent on AM fungi (Cheeke et al., 2019; Cheeke et al., 2021) and may not be able to establish in areas where the native mycorrhizal communities have been disrupted. Slow‐growing, late successional native prairie plant species often have strong dependence on AM fungi (Bauer et al., 2018; Koziol & Bever, 2015) and are also more sensitive to variation in AM fungal taxa than early successional native, or non‐native plant species (Cheeke et al., 2019; Koziol & Bever, 2016). Because many ecological restorations take place in soils with a legacy of invasion or repeated disturbances, such as in former agricultural fields, and AM fungi are dispersal limited (Delavaux et al., 2021; Tipton et al., 2022), it may be necessary to reintroduce a native AM fungal community at the same time as aboveground seeding or planting efforts (Koziol et al., 2018; Koziol & Bever, 2017).

However, inoculating every plant in a restoration or harvesting soil from remnant prairies to apply to disturbed landscapes is not practical or sustainable. One method of introducing the native soil community to restorations is to inoculate some plants with native soil organisms prior to transplanting to serve as a source of mycorrhizal inocula that can colonize other plants in the area. These inoculated seedlings—called “nurse plants”—have been used successfully in prairie restorations throughout the Midwestern United States (e.g., Koziol, Bauer, et al., 2022; Middleton & Bever, 2012). There is accumulating evidence that locally adapted soil biota are more effective for improving plant productivity than those in commercially produced inocula (Koziol, Schultz, et al., 2022; Lueck et al., 2024; Maltz & Treseder, 2015). This is because commercial inocula may contain only a few taxa or weedy taxa that have the potential to become invasive themselves (Jack et al., 2021), or not establish at all (Koziol et al., 2024; Koziol, McKenna, & Bever, 2025; Salomon et al., 2022). While AM fungal inoculations have been shown to benefit native plant growth in greenhouse (e.g., Bryant & Bever, 2024; Cheeke et al., 2019; Cheeke et al., 2021) and field experiments (House & Bever, 2020; Koziol, Bauer, et al., 2022; Koziol & Bever, 2017; Middleton et al., 2015), it is not clear (1) how the reintroduction of soil microbes into a restoration site may be impacted by site preparation technique, (2) whether or how long reintroduced microbes impact native plant growth and competitive ability over time, and (3) whether there may be additional benefits of inoculating with whole prairie soil containing a more diverse and complex microbial community compared to inoculating with AM fungal isolates.

The technique used for site preparation may be a strong selective force for determining whether microbes introduced via inoculations can establish and persist over time. Common site preparation techniques involve removing the existing vegetation via application of herbicide or through tillage (Smith et al., 2010), both of which can disturb above and belowground communities (e.g., Lekberg et al., 2017; Oehl et al., 2010; Oehl et al., 2017; Zaller et al., 2014). Although removing existing vegetation prior to planting or seeding may initially allow native species to establish more quickly, this additional disturbance may also create new opportunities for invasive species to take hold. Another strategy to eliminate the existing vegetation prior to planting is solarization (e.g., via tarping, Orr et al., 2019), which removes the existing vegetation but may have fewer negative effects on belowground organisms compared to herbicides or tilling. Alternatively, overseeding, when native species are seeded directly into existing vegetation, is a site preparation technique that minimizes disturbance to the restoration site because there is no physical (e.g., tillage) or chemical (e.g., herbicide) disruption to the above or belowground community. However, it is unknown how different site preparation techniques, with and without microbial inoculations, may impact native plant establishment in the restoration of invaded grasslands, especially over the long term.

Previous studies in the greenhouse (e.g., Bauer et al., 2018; Bryant & Bever, 2024; Cheeke et al., 2021) and field (e.g., Koziol et al., 2018; Koziol, Bauer, et al., 2022; Middleton & Bever, 2012) demonstrate the importance of native AM fungi in promoting the growth of some native plant species, including late successional native species of high conservation value (Cheeke et al., 2019; Koziol & Bever, 2015). However, most soil inoculation studies are on the order of months to a few years, and long‐term studies in the field, especially those over 5 years are largely absent from the literature. Furthermore, it is not known whether the benefits of inoculation with native AM fungi increase over time and/or whether there is a lag phase for the inoculated microbes to establish in a restoration site before differences in plant community composition are detected. Our long‐term field study tracks plant community composition over 7 years postinoculation and our findings provide practitioners with a strong, flexible framework for understanding when, where, and how soil microbial inoculations can be used in different site conditions, offering multiple pathways to restoration success.

**FIGURE 1:**
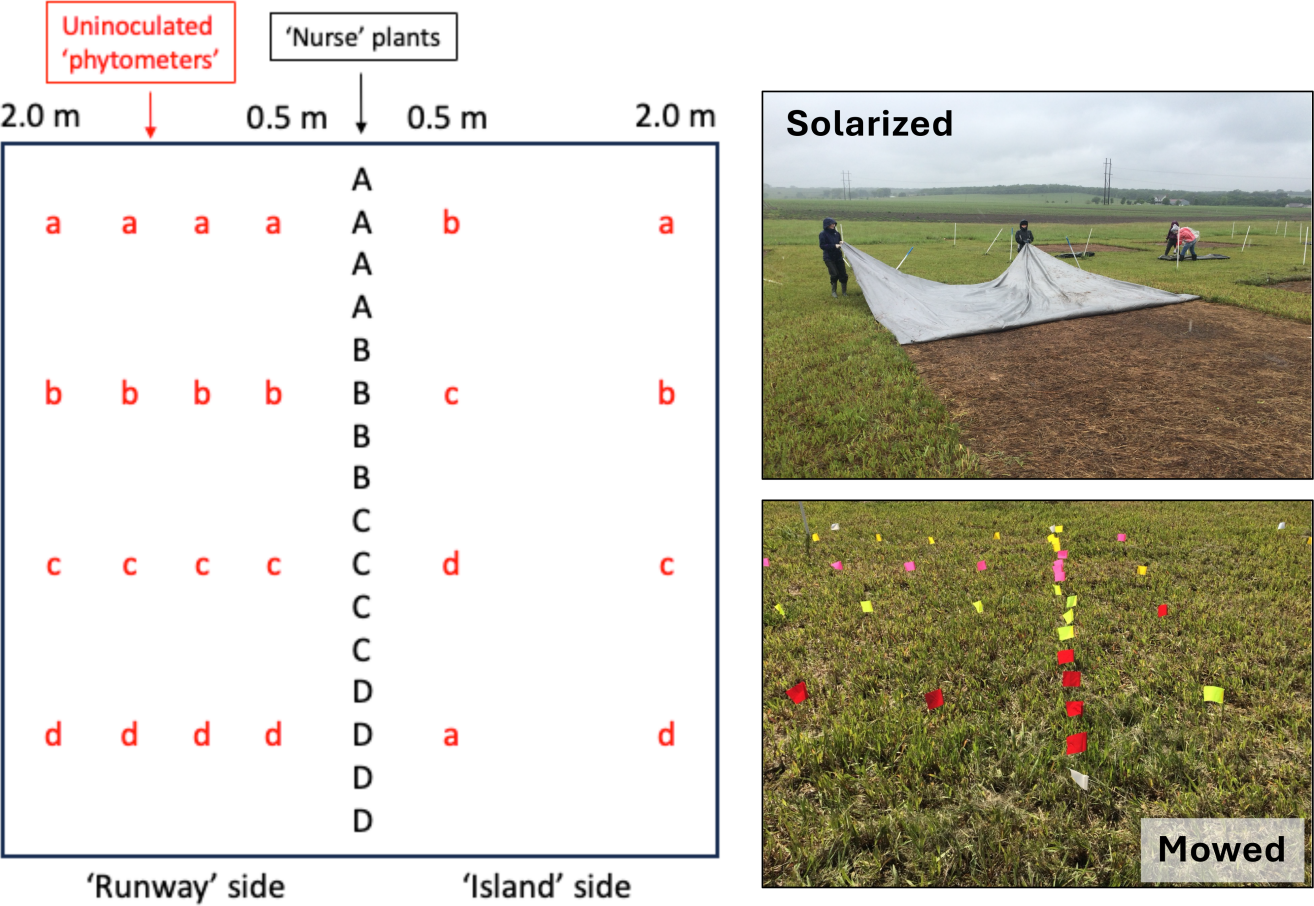
Plot design and site preparation treatment. Plot design (left) used to test for plant–soil feedbacks and the spread of microbes from the nurse plant row (black capital letters, center) to the uninoculated phytometer seedlings (red lower case letters). Nurse plants along the center row of each 5 × 5 m plot were (A) *Schizachyrium scoparium*, (B) *Echinacea pallida*, (C) *Lespedeza capitata*, and (D) *Asclepias tuberosa*, inoculated with either arbuscular mycorrhizal fungi, whole prairie soil, or uninoculated (Control). Treatments were applied at the plot level in a Latin Square Design, with six replicates of each for 36 plots in the experiment. Uninoculated phytometers (red) of the same four plant species (a, b, c, d) were planted at different distances on either side of the nurse plant row in each plot (0.5 m, 1.0 m., 1.5 m, 2.0 m). At the 0.5 m distance, on the “runway” side (left), phytometer seedlings were conspecific with the closest nurse plant species in the center row and on the “island” side (right), phytometer seedlings were heterospecific with the closest nurse plant species in the center row. The top photo shows the solarized plots at the time tarps were removed, and the bottom photo shows mowed plots. Both photos were taken at the time of planting and seeding in May 2016 (Lawrence, Kansas, USA, top photo by James D. Bever, bottom photo by Tanya E. Cheeke).

We tracked the development of native plant communities over seven growing seasons in plots that had been inoculated or not with local native soil biota, and using different site preparation techniques (solarized or mowed) as novel starting points. Specifically, we tested whether inoculating native tallgrass prairie seedlings with native soil microbes would improve native prairie plant establishment over time in the restoration of a non‐native *B. inermis* dominated grassland. Microbial inoculation treatments were introduced into field plots using four native species of “nurse plants”: a milkweed, *Asclepias tuberosa*, an aster, *Echinacea pallida*, a legume, *Lespedeza capitata*, and a C4 grass, *Schizachyrium scoparium*. Nurse plant seedlings were inoculated with local AM fungi that had been isolated and cultured from old‐growth remnant prairies, whole soil containing a suite of soil organisms from a nearby remnant prairie, or autoclaved inocula. To test the hypothesis that microbes from prairie remnants will improve native seedling survival, growth, and competitive ability in invaded grasslands, nurse plants were planted along the center of plots in which *B. inermis* was either eliminated via solarization or was mowed (Figure 1). To test for plant–soil feedbacks and to determine whether the introduced AM fungi migrated from the site of inoculation, uninoculated seedlings (hereafter called phytometers) were planted at different distances from the nurse plant row and their survival and growth were measured over time (Figure 1). Additionally, we tested whether site preparation technique, inoculation treatment, or their interactions impacted the development of a seeded plant community over seven growing seasons. Whether there was a differential benefit of using whole prairie soil inocula versus AM fungi was also tested.

## METHODS

### Study site

The field experiment took place at The Land Institute's Perennial Agriculture Field Station (Lawrence, Kansas, USA; 39°00′03.7″ N 95°19′13.9″ W) on a location that was historically prairie but was farmed for more than a hundred years. Prior to the experiment, the field site had been a cool‐season pasture dominated by non‐native *B. inermis* for at least 20 years. There was a conventionally managed agricultural field to the west, an old field to the north, a road and farm fields to the south, and a homestead to the east. The climate is characterized by cold, dry, windy winters and hot, wet, humid summers. The mean annual high temperature is 18.3°C, and the mean annual low temperature is 6.7°C; the mean annual precipitation is 101.3 cm year^−1^ (https://www.usclimatedata.com/climate/lawrence/kansas/united-states/usks0319). There was a drought that affected the region in 2018. Prescribed burns were applied to the field site in the winter of 2019, 2020, and 2021.

### Experimental design

This field restoration study consisted of two site preparation techniques (solarized or mowed) and three microbial inoculation treatments (AM fungi, whole prairie soil, autoclaved inocula) which were replicated six times in a Latin Square design, for a total of 36 plots (six, 5 m × 5 m plots arranged in six complete blocks; see field design, Appendix S1: Figure S1). There were 3‐m aisles between plots and field edges to limit microbial spread between plots. Each plot contained a center row of 16 “nurse” plant seedlings and 24 off‐center uninoculated phytometer seedlings (see plot design, Figure 1), for a total of 1440 seedlings in the experiment. After the nurse plant seedlings were planted, all plots were seeded with a diverse native seed mix (May 2016). Data on nurse plant and phytometer survival and growth were collected through the fourth growing season (2016–2019), and data on plant community composition were collected in the second growing season (2017), fourth growing season (2019), and seventh growing season (2022).

### Soil

#### Restoration site

The soil at the restoration site is classified as a Sharpsburg silt loam (parent material 100% loess). Data on soil nutrients and properties (Kansas State University Soil Testing Lab, Manhattan, KS, USA; Appendix S1: Table S1) were collected before soil inoculation treatments were added to get a baseline metric of soil nutrients in each plot. Four soil samples (10 cm depth) were collected from each plot in May 2016 and composited (one composite sample from each plot) for nutrient analysis (Appendix S1: Table S1).

#### Background sand/soil mix for nurse plant and phytometer seedlings

Soil for seedlings propagated in a greenhouse was collected from an old field near the field site (University of Kansas Field Station, Lawrence, KS, USA) and mixed 1:1 with Indiana river sand to improve drainage. The sand/soil mix was sterilized in an electric soil sterilizer (Model SS‐60; Pro‐Grow Supply, Brookfield, WI, USA) to eliminate resident soil organisms by heating to 82.2°C for 8 h, letting the soil cool for 24 h, and heating to 82.2°C again for 12 h. The heat‐treated 1:1 sand/soil mix was used as the background substrate for all seedlings in the experiment.

### Site preparation technique

The non‐native grass *B. inermis* was eliminated from 18 field plots via solarization with large, heavy‐duty poly black, non‐transparent, waterproof tarps (TRP, Inc.). The 18 solarized plots were covered with tarps from September 2015 to May 2016 and the other 18 field plots, as well as edges and aisles, were mowed just prior to planting in May 2016 (see field design in Appendix S1: Figure S1).

### Inocula

The AM fungal inocula treatment consisted of a mixture of seven native AM fungal taxa that were isolated from old‐growth remnant prairies in Kansas and Missouri, USA, (Appendix S1: Table S2) and cultured on mixed native plant species from July 2014 to March 2015 in a greenhouse at Indiana University (Bloomington, IN, USA). AM fungi were cultured using the Single Species Culture method described by the International Collection of Vesicular Arbuscular Mycorrhizal Fungi (INVAM) (https://invam.ku.edu/single-species-cultures). Briefly, spores of AM fungi were extracted from remnant prairie soil using sucrose density‐gradient centrifugation, separated by species using a Pasteur pipette and dissecting microscope, and monospecific AM fungal cultures were grown with native plant seedlings to generate spores. The seven different spore cultures (*Claroideoglomus clariodium, Glomus geosporum, Acaulospora morrowiae, Gigaspora gigantea, Acaulospora koskei, Scutellospora dipurpurascens, Funneliformis mosseae*) were mixed together by volume for the arbuscular mycorrhizal fungi (AMF) treatment used in this experiment (Appendix S1: Table S2). The whole soil inocula treatment consisted of local prairie remnant soil collected from Rockefeller Prairie in late February 2016, Lawrence, KS, USA, (7.0 ppm P Bray‐1 equivalent, 106 ppm K, 2 ppm NO_3_‐N, 8 ppm NH_4_‐N, pH 5.9; Silt loam, 9% sand, 64% silt, 27% clay) and mixed 1:1 with autoclaved Indiana river sand.

### Seedlings

Nurse plants and phytometer seedlings were propagated in a greenhouse at Indiana University (Bloomington, IN, USA) in spring 2016. Four native tallgrass prairie plant species, *Asclepias tuberosa* (Prairie Moon Nursery, Winona, MN, USA), *Echinacea pallida* (Missouri Wildflowers, Jefferson City, MO, USA), *Lespedeza capitata* (Missouri Wildflowers, Jefferson City, MO, USA), and *Schizachyrium scoparium* (Missouri Wildflowers, Jefferson City, MO, USA) were selected for their dependence on AM fungi, their mid to late successional perennial life history status, their importance in native prairie ecosystems, and the resources they provide for local pollinators. Seeds were cold‐moist stratified (*E. pallida, A. tuberosa*), scarified (*L. capitata*), or received no preparation treatment (*S. scoparium*) and germinated in autoclaved potting media (Canadian Sphagnum Peat Moss, Bark, Vermiculite, Dolomite Lime, Long‐Lasting Wetting Agent, RESiLIENCE; Metro‐Mix 360, SunGro Horticulture, Agawam, MA, USA) for approximately 1 month before transplanting into inoculation treatments.

### Nurse plant inoculation and preparation of phytometers

In March 2016, nurse plant seedlings were transplanted into conetainers (Stuewe and Sons, Tangent, OR, USA) that contained 125 cc of sterilized background sand/soil mix and inoculated at a rate of 17% by volume, with either AM fungal inocula, whole prairie soil, or autoclaved inocula (uninoculated controls) (Appendix S1: Table S2). Each conetainer contained 62.5 cc of sterilized sand/soil mix at the bottom, 25 cc of inocula treatment in the middle, and 62.5 cc of sterilized sand/soil mix at the top. This “sandwich” inoculation technique places the inocula at the root zone and reduces the chance of contamination of pots via dust or splashing. Uninoculated phytometers (the same four species as the nurse plants) were transplanted into conetainers containing 150 cc of sterilized background sand/soil mix. Seedlings were grown in their respective treatments in a greenhouse (Indiana University, Bloomington, IN, USA) for 1 month prior to being hardened off and transplanted into the field. Absence of AM fungal colonization in uninoculated seedlings and presence of AM fungal colonization in inoculated seedlings was confirmed via microscopy.

### Transplanting into field plots

In May 2016, 40 native prairie seedlings were transplanted into each plot, including 16 nurse plant seedlings and 24 uninoculated phytometers (40 seedlings per plot × 36 plots = 1440 seedlings). Four individual nurse plants of each of the four nurse plant species, *A. tuberosa*, *E. pallida*, *L. capitata*, and *S. scoparium* were planted in a row in the center of each plot, with 0.25‐m spacing between each plant (Figure 1). The four replicate seedlings of each nurse plant species were planted together along the center row (Figure 1), with the planting order of each species randomized in each plot. Each plot received a single inoculation treatment, with 16 nurse plants inoculated with the AM fungal treatment in the “AMF” plots, 16 nurse plants inoculated with whole prairie soil in the “whole soil” plots, and 16 uninoculated nurse plants in the “control” plots. Each seedling was marked at the base with a metal tag so that the same plant was observed at each data collection point.

### Test of microbial migration and plant soil feedbacks

To investigate how far AM fungi and other microbes spread from the nurse plant row into the field plots, uninoculated prairie seedlings, called phytometers, were planted at varying distances from the inoculated nurse plant row (Figure 1). On one side of each plot, phytometer seedlings were planted 0.5, 1.0, 1.5, and 2.0 m from the nurse plant row (hereafter called the “runway” side; Figure 1) and on the other side of each plot, phytometer seedlings were planted 0.5 m and 2.0 m from the nurse plant row (hereafter called the “island” side; Figure 1). The placement of the “runway” versus “island” phytometer seedlings alternated between each block so that the “runway” was on the south side of the nurse plant row in Blocks 1, 3, and 5, and on the north side of the nurse plant row in Blocks 2, 4, and 6 to account for environmental variation in the field. The phytometer seedling “runway” was designed to test whether a row of uninoculated native seedlings would facilitate the spread of AM fungi from inoculated nurse plants in the center of the plot into the restoration area more efficiently than phytometers planted with greater spacing between them on the “island” side (Figure 1). To test for plant–soil feedbacks, that is, differential effects of neighbors mediated by microbes (Bever et al., 1997) that could impact native plant establishment, the uninoculated phytometers planted on the “runway” side were conspecific with the closest nurse plants in the center row, but on the “island” side, phytometers planted closest to the nurse plant row (0.5 m distance) were heterospecific to the nurse plant species but conspecific at the 2.0‐m distance (Figure 1). Plant soil feedback effects were tested as the interaction of nurse plant identity and inoculation on performance at the 0.5 m distance.

### Diverse prairie seed mix

To test for effects of site preparation technique, microbial inoculation, and their interactions on the development of the plant community, all plots were seeded with a diverse native prairie seed mix (Appendix S1: Table S3) in May 2016 after the nurse plant and phytometer seedlings were transplanted into plots. The diverse seed mix consisted of local ecotypes of 39 plant species (approximately 60:40 mix of native forbs:grasses), which was prepared as follows: 1.36 kg of Diverse “Deep Soil” seed mix (Missouri Wildflowers, Jefferson City, MO, USA), which consisted of 33 native forb species, 0.23 kg each of the native grasses *S. scoparium, Andropogon gerardii* (big bluestem), *Bouteloua curtipendula* (sideoats grama), *Sorghastrum nutans* (Indiangrass), and 14.17 g each of the milkweeds *Asclepias syriaca* (common milkweed) and *A. incarnata* (swamp milkweed) (Missouri Wildflowers, Jefferson City, MO, USA; Appendix S1: Table S3). Plant species in the diverse prairie seed mix were chosen based on recommendations from local restoration practitioners, academic researchers, native seed companies, plant successional stage, and their importance in restorations and to local pollinators. Seeding rates for plots and aisles were based on recommendations from Missouri Wildflowers and local restoration practitioners. The seeds were mixed with autoclaved sand and cold‐moist stratified before being applied at a rate of 15 kg ha^−1^ in plots and 5 kg ha^−1^ in aisles and field edges. Seeds were applied directly to bare soil in the solarized plots and were overseeded into the mowed plots, aisles, and field edges. The field site was seeded again in November 2016, with the same seed mix at the same rate (Appendix S1: Table S3). In February 2021, an additional seed mix containing 18 prairie species was added at a rate of 225 seeds species^−1^ plot^−1^ (Appendix S1: Table S3).

### Data collection

Data on nurse plant and phytometer seedling survival and growth (e.g., height) were collected every year over the first four growing seasons without destructive sampling. Plant community composition data were recorded in the second growing season (June 2017), the fourth growing season (August 2019), and the seventh growing season (August 2022). Because of the labor involved in measuring every transplanted seedling in the restoration, in 2022, only plant community composition data were collected from each plot. Here, we focus on the results of the final plant community composition collected in the seventh growing season (summer 2022). To do this, we recorded the percent cover of each plant species observed in a 1‐m^2^ quadrat that had been placed in the center of each plot. Percent cover data were used to determine how site preparation technique, microbial inoculation, and/or their interactions impacted the development of the final plant community. We also used the percent cover data to determine how other metrics, such as Shannon diversity, mean floristic quality index (FQI), and relative abundances of, for example, native versus non‐native species; early versus late successional plant species may have varied across the different experimental treatments. A summary of results from plant community data collected in June 2017 and August 2019 are reported in Appendix S1: Results S1.

### Data analysis

Analyses were performed using proc mixed and proc glimmix in SAS and in R (version 4.5.1).

#### Tests of nurse plant survival and growth

Mixed models were used to analyze nurse plant growth with initial height at transplant, block, site preparation technique, inoculation treatment, plant species, site preparation × inoculation interactions, plant species × inoculation interactions, plant species × site preparation technique interactions, and species × inoculation × site preparation interactions. To compare the effects of the different inoculation treatments on nurse plant survival and growth, orthogonal a priori contrasts were designed to compare plant growth with living inocula (whole soil and AM fungal cultures) versus non‐inoculated seedlings, growth difference when inoculated with AM fungi versus whole prairie soil, and these contrasts by site preparation technique. Marginal means and standard errors for survival data were back‐transformed from model estimates for data visualization. Raw means were used to visualize growth of the surviving nurse plant seedlings over time, as many seedlings died over the course of the experiment and marginal means were not always able to be estimated.

#### Test of plant–soil feedbacks and migration of AM fungi into the field site

Plant soil feedback effects were tested as the interaction of nurse plant species identity and inoculation on performance of the uninoculated phytometers planted at the 0.5 m distance from the nurse plant row. Mixed models for these analyses included block, plant species, site preparation treatment, inoculation treatment, and whether the phytometer species were conspecific or heterospecific to the closest nurse plant.

#### Plant community composition

To visualize differences in plant community composition among site preparation techniques and inoculation treatments, we used principal coordinates analysis (PCoA) using Bray–Curtis distance matrices calculated from relative abundances of plant species in each plot during the second (June 2017), fourth (August 2019), and seventh growing (August 2022) seasons. Permutational multivariate analysis of variance (PERMANOVA), using the adonis2 function in R, was used to test for significant differences in species composition among site preparation techniques, inoculation treatments, and their interactions. Homogeneity of multivariate dispersion was assessed using permutational multivariate analysis of dispersion (PERMDISP). We analyzed differences between plot vegetation (e.g., relative abundance of non‐native plant species, FQI, and mean coefficient of conservatism [CC]) (Missouri region, 2015) in a general linear model with site preparation technique, inoculation treatment, the interaction of these two factors, and experimental block as fixed effects.

## RESULTS

### The effect of inoculation and vegetation removal on transplanted nurse plant growth, survival, and neighboring phytometers

#### Inoculation with soil microbes improved native nurse plant seedling survival

**FIGURE 2:**
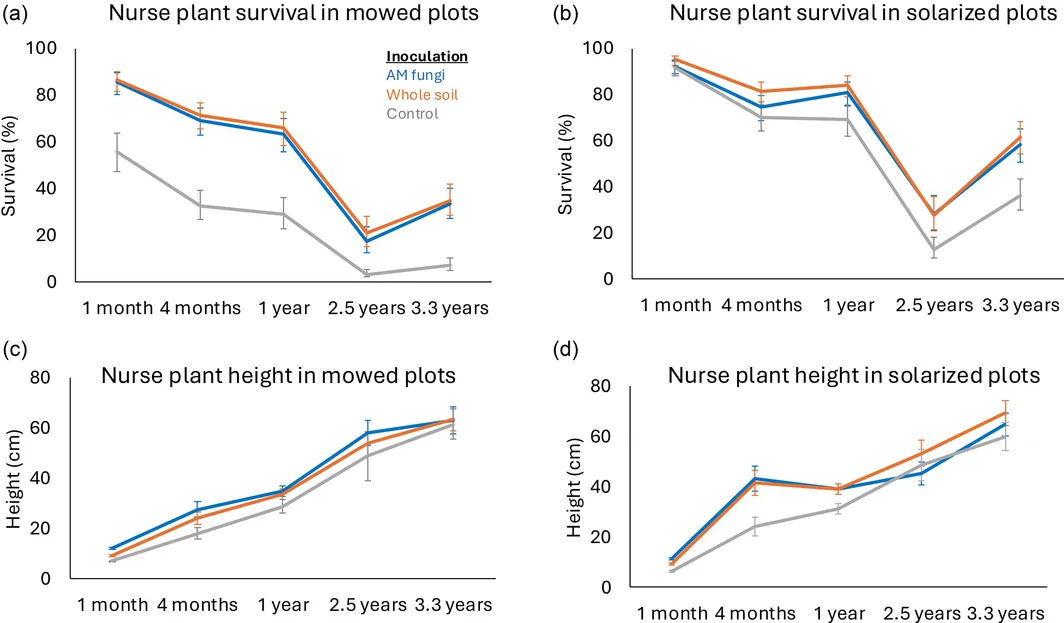
Nurse plant seedling survival (a, b) and growth (c, d) 1 month (June 2016), 4 months (September 2016), 1 year (June 2017, second growing season), 2.5 years (October 2018, third growing season), and 3.3 years (August 2019, fourth growing season) after being transplanted into mowed (a, c) or solarized plots (b, d) (Lawrence, KS, USA). Native prairie nurse plant seedlings (*Asclepias tuberosa, Echinacea pallida*, *Lespedeza capitata*, and *Schizachyrium scoparium*) were inoculated with either local arbuscular mycorrhizal fungi (AMF, blue), whole soil from a nearby remnant prairie (whole soil, orange), or remained uninoculated (control, gray) and planted into field plots that had been either solarized (*n* = 18 solarized plots) to eliminate the existing cover of *Bromus inermis* or mowed (*n* = 18 mowed plots) prior to planting and seeding in May 2016.

Inoculation with native soil microbes improved the survival of nurse plant seedlings over four growing seasons (Figure 2a,b; Table 1). Although native seedling survival was higher overall in the solarized plots (Figure 2b), the benefits of inoculation were more apparent in the mowed plots (Figure 2a), where seedlings were in direct competition with the non‐native grass, *B. inermis*. There were no differences in survival between nurse plants inoculated with AM fungi versus whole prairie soil in either mowed or solarized plots (Figure 2a,b; AMF vs. whole soil contrast, Table 1), demonstrating that the AM fungi and whole soil microbial inoculation treatments were functionally equivalent in this study. Survival rates did not vary across the four nurse plant species, except that in the second growing season, *S. scoparium* had higher survival compared to the other species (Table 1, *p* = 0.02; Appendix S1: Figure S2).

**TABLE 1:** Type III test of fixed effects for nurse plant survival 1 month (June 2016), 4 months (September 2016), 1 year (June 2017), 2.5 years (October 2018), and 3.3 years (August 2019) after being transplanted into field plots (Lawrence, KS, USA).

Nurse plant survival effect	Num DF	Den DF	*F* value	*p* value
First growing season (June 2016)				
Initial height	4	529	1.08	0.3663
Block	5	529	0.49	0.7869
Tarp	1	25	8.96	**0.0061**
Inoc	2	25	3.74	**0.038**
*live* versus *control*	1	25	6.56	**0.0169**
*AMF* versus *whole*	1	25	0.9	0.353
Tarp × Inoc	2	25	2.14	0.1384
*live* versus *control* × *tarp*	1	25	3.86	0.0607
*AMF* versus *whole soil* × *tarp*	1	25	0.34	0.5675
Plant Species	3	529	1.35	0.257
Plant species × tarp	3	529	1.05	0.3709
Plant species × Inoc	.	.	.	.
First growing season (September 2016)				
Initial height	4	529	1.86	0.1154
Block	5	529	1.61	0.1546
Tarp	1	25	6.44	**0.0178**
Inoc	2	25	8.63	**0.0014**
*live* versus *control*	1	25	14.05	**0.0009**
*AMF* versus *whole*	1	25	2.1	0.1593
Tarp × Inoc	2	25	4.58	**0.0429**
*live* versus *control* × *tarp*	1	15	6.76	**0.0155**
*AMF* versus *whole soil* × *tarp*	1	15	0.45	0.5063
Plant Species	3	529	0.53	0.6602
Plant species × tarp	3	529	0.99	0.3954
Plant species × Inoc	.	.	.	.
Second growing season (June 2017)				
Initial height	4	523	3.3	**0.0109**
Block	5	523	0.75	0.5878
Tarp	1	25	12.96	**0.0014**
Inoc	2	25	7.75	**0.0024**
*live* versus *control*	1	25	13.16	**0.0013**
*AMF* versus *whole*	1	25	3.34	0.0794
Tarp × Inoc	2	25	0.89	0.422
*live* versus *control* × *tarp*	1	25	1.48	0.2356
*AMF* versus *whole soil* × *tarp*	1	25	0.25	0.621
Plant Species	3	523	3.23	**0.0222**
Plant species × tarp	3	523	2.59	**0.0521**
Plant species × Inoc	6	523	1.9	0.0788
Third growing season (October 2018)				
Initial height	4	529	2.04	0.0877
Block	5	529	1.01	0.4132
Tarp	1	25	2.39	0.1343
Inoc	2	25	7.73	**0.0024**
*live* versus *control*	1	25	13.23	**0.0012**
*AMF* versus *whole*	1	25	1.22	0.2796
Tarp × Inoc	2	25	1.2	0.3176
*live* versus *control* × *tarp*	1	25	2.26	0.1457
*AMF* versus *whole soil* × *tarp*	1	25	0.12	0.7371
Plant Species	3	529	0.29	0.8302
Plant species × tarp	3	529	1.31	0.2697
Plant species × Inoc	.	.	.	.
Fourth growing season (August 2019)				
Initial height	4	523	2.87	**0.0227**
Block	5	523	1.69	0.135
Tarp	1	25	20.33	**0.0001**
Inoc	2	25	9.55	**0.0008**
*live* versus *control*	1	25	16.5	**0.0004**
*AMF* versus *whole*	1	25	2.21	0.1496
Tarp × Inoc	2	25	2.73	0.0845
*live* versus *control* × *tarp*	1	25	5.14	**0.0322**
*AMF* versus *whole soil* × *tarp*	1	25	0.24	0.6275
Plant Species	3	523	0.22	0.8835
Plant species × tarp	3	523	5.54	**0.001**
Plant species × Inoc	6	523	0.95	0.4559

*Note*: Native prairie nurse plant seedlings (*Asclepias tuberosa*, *Echinacea pallida*, *Lespedeza capitata*, *Schizachyrium scoparium*) were grown in the following inoculation treatments (Inoc; arbuscular mycorrhizal fungi, AMF; whole prairie soil, whole; or no inocula, control) and were planted along the center of each solarized or mowed plot (Tarp effect indicates solarized or mowed). Pair‐wise contrasts among inoculation treatments are shown in italics. “Live” represents plants inoculated with AMF or whole prairie soil, combined into one “live” soil treatment for analysis, and “control” represents the uninoculated plants. *p* values in bold indicate significant effects.

Abbreviation: DF, degrees of freedom.

Inoculation was especially important for the survival of nurse plants in the mowed plots within the first year of the study, where survival of inoculated seedlings was >30% higher than seedlings that had not been inoculated (Figure 2a). Most of the seedlings that survived the first 4 months of the study in both solarized and mowed plots were still alive the following year, and inoculation increased their chance of survival (Figure 2a,b). Nurse plant survival appeared to decline 2.5 years after planting due to drought and a later sampling date (October 2018), but many plants recovered by the following year (Figure 2a,b), likely because of their perennial nature and deep root systems. By the fourth growing season (3.3 years after planting), 35% of the inoculated seedlings survived in the mowed plots (Figure 2a) compared to >60% of inoculated seedlings in the solarized plots (Figure 2b). In both mowed and solarized plots, most of the uninoculated seedlings died by the fourth growing season, with 7% survival of uninoculated seedlings in the mowed plots (Figure 2a) and 35% survival of uninoculated seedlings in the solarized plots 3.3 years after planting (Figure 2b).

#### Inoculation improved native nurse plant seedling growth, especially during the drought year

Nurse plant seedlings grew taller when inoculated with prairie microbes (Figure 2c,d), particularly in the solarized plots within the first year of the study (Figure 2d; Table 2). However, the benefits of inoculation on plant growth varied by plant species (Species × Inoc interactions, Table 2; Appendix S1: Figure S3). The C4 native grass, *S. scoparium* and the native legume *L. capitata* generally benefitted from microbial inoculations compared to uninoculated controls over the course of the study (Appendix S1: Figure S3). In the drought year (2.5 years after transplanting; October 2018), nurse plants inoculated with whole prairie soil were taller than plants inoculated with AM fungi (Figure 2d; AMF vs. whole soil contrast, *F*_1,25_ = 13.28, *p* = 0.0013; Table 2), which appears to be driven by the strong increase in growth response of the native legume *L. capitata* to the whole soil treatment in the year that water was limited (Appendix S1: Figure S3).

**TABLE 2:** Type III test of fixed effects for nurse plant height (cm) 4 months (September 2016), 1 year (June 2017), 2.5 years (October 2018), and 3.3 years (August 2019) after being transplanted into field plots (Lawrence, KS, USA).

Effect	Num DF	Den DF	*F* value	*p* value
September 2016 (4 months)				
Initial height	4	295	3.53	**0.0078**
Block	5	295	0.94	0.4451
Tarp	1	25	31.26	**<0.0001**
Inoc	2	25	13.85	**<0.0001**
*live* versus *control*	1	25	27.44	**<0.0001**
*AMF* versus *whole soil*	1	25	0.06	0.8081
Tarp × Inoc	2	25	0.29	0.7518
*live* versus *control* × *tarp*	1	25	0.22	0.6434
*AMF* versus *whole soil* × *tarp*	1	25	0.36	0.5548
Species	3	295	6.89	**0.0002**
Species × Inoc	6	295	3.58	**0.0019**
Species × Tarp	3	295	2.05	0.1068
Species × Tarp × Inoc	6	295	1.70	0.1218
June 2017 (1 year)				
Initial height	4	299	0.76	0.5517
Block	5	299	0.73	0.6043
Tarp	1	25	13.42	**0.0012**
Inoc	2	25	3.93	**0.0328**
*live* versus *control*	1	25	7.82	**0.0098**
*AMF* versus *whole soil*	1	25	0.00	0.9695
Tarp × Inoc	2	25	0.15	0.8634
*live* versus *control* × *tarp*	1	25	0.06	0.8088
*AMF* versus *whole soil* × *tarp*	1	25	0.23	0.6321
Species	3	299	1.46	0.2263
Species × Inoc	6	299	3.38	**0.0031**
Species × Tarp	3	299	2.77	**0.0421**
Species × Tarp × Inoc	6	299	1.69	0.1277
Oct. 2018 (2.5 years)	3	113	7.40	**0.0001**
Initial height	5	113	0.81	0.5427
Block	1	24	0.00	1.0000
Tarp	2	24	8.70	**0.0014**
Inoc	.	.	.	.
*live* versus *control*	1	24	13.28	**0.0013**
*AMF* versus *whole soil*	2	24	1.76	0.1943
Tarp × Inoc	.	.	.	.
*live* versus *control* × *tarp*	1	24	2.17	0.1542
*AMF* versus *whole soil* × *tarp*	1	113	0.00	1.0000
Species	4	113	2.75	**0.0318**
Species × Inoc	3	113	2.11	0.1029
Species × Tarp	3	113	2.08	0.1065
Species × Tarp × Inoc				
Aug. 2019 (3.3 years)				
Initial height	4	184	4.66	**0.0013**
Block	5	184	3.15	**0.0095**
Tarp	1	25	5.36	**0.0290**
Inoc	2	25	15.58	<**0.0001**
*live* versus *control*	.	.	.	.
*AMF* versus *whole soil*	.	.	.	.
Tarp × Inoc	2	25	0.10	0.9023
*live* versus *control* × *tarp*	.	.	.	.
*AMF* versus *whole soil* × *tarp*	.	.	.	.
Species	3	184	7.58	**<0.0001**
Species × Inoc	6	184	2.56	**0.0209**
Species × Tarp	3	184	0.07	0.9756
Species × Tarp × Inoc	3	184	0.97	0.4098

*Note*: Native prairie nurse plant seedlings (Species; *Asclepias tuberosa, Echinacea pallida*, *Lespedeza capitata*, and *Schizachyrium scoparium*) were grown in the following inoculation treatments (Inoc; arbuscular mycorrhizal fungi, AMF; whole prairie soil, whole soil; or no inocula, control) and were planted along the center of each solarized or mowed plot (Tarp effect indicates solarized or mowed). Pair‐wise contrasts among inoculation treatments are shown in italics. “Live” represents plants inoculated with AMF or whole prairie soil, combined into one “live” soil treatment for analysis, and “control” represents the uninoculated plants. *p* values in bold indicate significant effects.

#### Evidence of impact of inoculation on neighboring phytometers

We used uninoculated native prairie seedlings (phytometers) planted at different distances (0.5–2.0 m) from the nurse plant row (Figure 1) to test for plant–soil feedbacks that may improve or limit the survival or growth of native seedlings in the field over time. Four months after transplanting (September 2016), phytometers that were planted 0.5 m from the nurse plant row had higher survival when grown near nurse plants with the AM fungal inocula compared to those grown near nurse plants inoculated with whole prairie soil (Appendix S1: Figure S4a), and effects were stronger in the mowed compared to the solarized plots (Appendix S1: Figure S4b), as shown by a solarization by inoculation effect (*p* = 0.0545; Table 3). However, this effect faded over time (Table 3). In the drought year of 2018, survival of phytometers was higher in the whole soil inoculation treatment compared to the AM fungal treatment in mowed, but not solarized plots (*F*_1,25_ = 4.63, *p* = 0.0412; Table 3), but this effect was not detected the following year (*F*_1,25_ = 0.20, *p* = 0.6550; Table 3). We did note that survival or size of uninoculated phytometers was affected by the species identity of their neighboring nurse plant over the first three growing seasons (Table 3, “species”), as would result from differential plant soil feedbacks (Bever et al., 1997).

**TABLE 3:** Test of plant soil feedbacks based on the survival of uninoculated phytometer seedlings 0.5 m from the nurse plant row from June 2016 through August 2019.

Effect	Num DF	Den DF	*F* value	*p* value
June 2016 (1 months)				
Initial height	4	238	0.63	0.6451
Block	5	238	0.90	0.4816
Tarp	1	25	21.15	**0.0001**
Inoc	2	25	0.39	0.6796
*live* versus *control*	1	25	0.43	0.5174
*AMF* versus *whole soil*	1	25	0.39	0.5388
Tarp × Inoc	2	25	0.83	0.4475
*live* versus *control* × *tarp*	1	25	1.37	0.2527
*AMF* versus *whole soil* × *tarp*	1	25	0.36	0.5524
Conspecific	1	238	0.14	0.7055
Tarp × Conspecific	1	238	0.67	0.4149
Inoc × Conspecific	2	238	1.25	0.2875
Tarp × Inoc × Conspecific	2	238	1.31	0.2714
Species	3	238	0.86	0.4607
Sept. 2016 (4 months)				
Initial height	4	238	1.15	0.3356
Block	5	238	0.86	0.5074
Tarp	1	25	20.42	**0.0001**
Inoc	2	25	1.94	0.1652
*live* versus *control*	1	25	0.34	0.5636
*AMF* versus *whole soil*	1	25	3.56	**0.0709**
Tarp × Inoc	2	25	3.28	**0.0545**
*live* versus *control* × *tarp*	1	25	5.45	**0.0279**
*AMF* versus *whole soil* × *tarp*	1	25	1.02	0.3219
Conspecific	1	238	1.53	0.2169
Tarp × Conspecific	1	238	0.00	0.9806
Inoc × Conspecific	2	238	0.27	0.7606
Tarp × Inoc × Conspecific	2	238	1.02	0.3638
Species	3	238	2.13	**0.0975**
June 2017 (1 year)				
Initial height	4	238	1.93	0.1066
Block	5	238	0.29	0.9158
Tarp	1	25	27.57	**<0.0001**
Inoc	2	25	1.57	0.2280
*live* versus *control*	1	25	0.07	0.7867
*AMF* versus *whole soil*	1	25	3.06	**0.0923**
Tarp × Inoc	2	25	0.78	0.4709
*live* versus *control* × *tarp*	1	25	1.32	0.2622
*AMF* versus *whole soil* × *tarp*	1	25	0.22	0.6436
Conspecific	1	238	0.33	0.5666
Tarp × Conspecific	1	238	0.65	0.4202
Inoc × Conspecific	2	238	0.58	0.5632
Tarp × Inoc × Conspecific	2	238	0.91	0.4058
Species	3	238	4.26	**0.0059**
Oct. 2018 (2.5 years)				
Initial height	4	238	0.42	0.7923
Block	5	238	1.45	0.2071
Tarp	1	25	17.60	**0.0003**
Inoc	2	25	2.26	0.1256
*live* versus *control*	1	25	1.81	0.1904
*AMF* versus *whole soil*	1	25	1.94	0.1761
Tarp × Inoc	2	25	2.34	0.1174
*live* versus *control* × *tarp*	1	25	0.03	0.8550
*AMF* versus *whole soil* × *tarp*	1	25	4.63	**0.0412**
Conspecific	1	238	0.01	0.9146
Tarp × Conspecific	1	238	0.34	0.5620
Inoc × Conspecific	2	238	0.02	0.9754
Tarp × Inoc × Conspecific	2	238	0.47	0.6242
Species	3	238	2.20	**0.0887**
Aug. 2019 (3.3 years)				
Initial height	4	238	0.12	0.9768
Block	5	238	0.80	0.5514
Tarp	1	25	16.87	**0.0004**
Inoc	2	25	0.13	0.8797
*live* versus *control*	1	25	0.00	0.9574
*AMF* versus *whole soil*	1	25	0.26	0.6180
Tarp × Inoc	2	25	0.72	0.4950
*live* versus *control* × *tarp*	1	25	1.23	0.2782
*AMF* versus *whole soil* × *tarp*	1	25	0.20	0.6550
Conspecific	1	238	0.75	0.3866
Tarp × Conspecific	1	238	0.00	0.9663
Inoc × Conspecific	2	238	0.16	0.8558
Tarp × Inoc × Conspecific	2	238	0.48	0.6216
Species	3	238	0.71	0.5482

*Note*: Bold indicates *p* < 0.10. Nurse plants, *Schizachyrium scoparium*, *Echinacea pallida*, *Lespedeza capitata*, and *Asclepias tuberosa* were inoculated with either arbuscular mycorrhizal fungi, whole prairie soil, or remained uninoculated and planted down the center of each plot (5 × 5 m). Uninoculated phytometers of the same four plant species were planted at different distances from the nurse plant row (0.5 m, 1.0 m., 1.5 m, 2.0 m) to test for plant–soil feedbacks and potential spread of inocula into the field site over time (see plot design in Figure 1). Phytometer seedlings were either conspecific or heterospecific with the nearest nurse plant species.

#### High mortality of uninoculated phytometers limited our ability to test for AM fungal migration over time

Because of the overall high mortality of the uninoculated phytometers in the field, we were unable to use the “runways” and “islands” we had originally constructed to statistically test for potential migration of soil microbes away from the nurse plant row. For example, a stronger growth response of phytometers planted near an inoculated nurse plant compared to phytometers planted near an uninoculated nurse plant would have suggested that the microbes migrated from the site of inoculation into the field plot over time. However, the poor survival of the uninoculated phytometers was consistent with the high mortality rates of the uninoculated nurse plants in the same plots (Figure 2a,b), highlighting the importance of native soil microbes to the survival of native prairie seedlings in the field.

### The effect of inoculation and solarization on overall plant community composition including native and non‐native plant cover and floristic quality

#### Site preparation technique and microbial inoculations steered the development of the plant community over time

**FIGURE 3:**
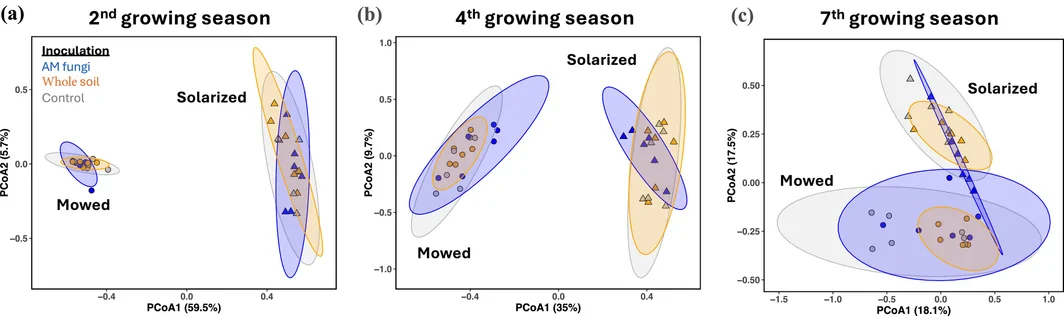
Principal coordinates analysis (PCoA) showing variation in plant community structure in (a) the second growing season (June 2017), (b) the fourth growing season (August 2019), and (c) the seventh growing season (August 2022) in field plots inoculated with arbuscular mycorrhizal fungi (AMF, blue), whole prairie soil (whole soil, orange) or remained uninoculated (Control, gray). Field plots had been either initially solarized (*n* = 18 solarized plots) to eliminate the existing cover of *Bromus inermis* or initially mowed (*n* = 18 mowed plots) prior to planting and seeding with a diverse seed mix in May 2016.

In the second growing season (June 2017), there were strong effects of the initial site preparation technique (mowed or solarized; PERMANOVA *R* ^2^ = 0.72, *p* = 0.001), but no effects of inoculation (*R* ^2^ = 0.01, *p* = 0.54) or their interactions (*R* ^2^ = 0.02, *p* = 0.45) on plant community structure (Figure 3a). In the fourth growing season (August 2019), there were strong effects of site preparation technique (PERMANOVA *R* ^2^ = 0.53, *p* = 0.001), but still no significant effects of inoculation (*R* ^2^ = 0.03, *p* = 0.39) or their interactions (*R* ^2^ = 0.02, *p* = 0.56) on plant community structure (Figure 3b), although the PCoA visualization shows that the plant communities were starting to become more similar in the mowed and solarized plots that had been initially inoculated with AM fungi (Figure 3b). By the end of the seventh growing season (August 2022), there were legacy effects of both site preparation technique (*R* ^2^ = 0.21, *p* = 0.001) and microbial inoculation (*R* ^2^ = 0.09, *p* = 0.020) on plant community structure (Figure 3c; Appendix S1: Table S4), but no site preparation technique by inoculation interactions was detected (*R* ^2^ = 0.046 = 1.04, *p* = 0.390; Appendix S1: Table S4). By the seventh growing season, the composition of the plant communities that received the AM fungal treatment in both the mowed and solarized plots began to converge (Figure 3c), whereas the plots that received the whole prairie soil treatment or were uninoculated maintained distinct plant communities between the solarized and mowed plots (Figure 3c).

#### Solarized plots and inoculated plots had a more desirable and diverse native plant community

In the seventh growing season, we observed 27 of the original 39 native plant species (69%) that had been seeded or planted in 2016, and 28% of species from the later seeding date in 2021 (Appendix S1: Table S3). The proportion of desirable native plant species, i.e., those that were intentionally seeded or planted into the restoration, was greater in the initially solarized plots (mean = 80.9% ± 3.3% relative cover) compared to the initially mowed plots (mean = 67.4% ± 3.3% relative cover; *F*_1,25_ = 8.24, *p* = 0.008; Appendix S1: Table S5). The proportion of desirable plant species was also greater in the inoculated plots (mean 80.6% ± 2.9% relative cover) compared to the uninoculated plots (mean = 61.2% ± 7.2% relative cover; *F*_2,25_ = 7.67, *p* = 0.0025; Appendix S1: Table S5).

**FIGURE 4:**
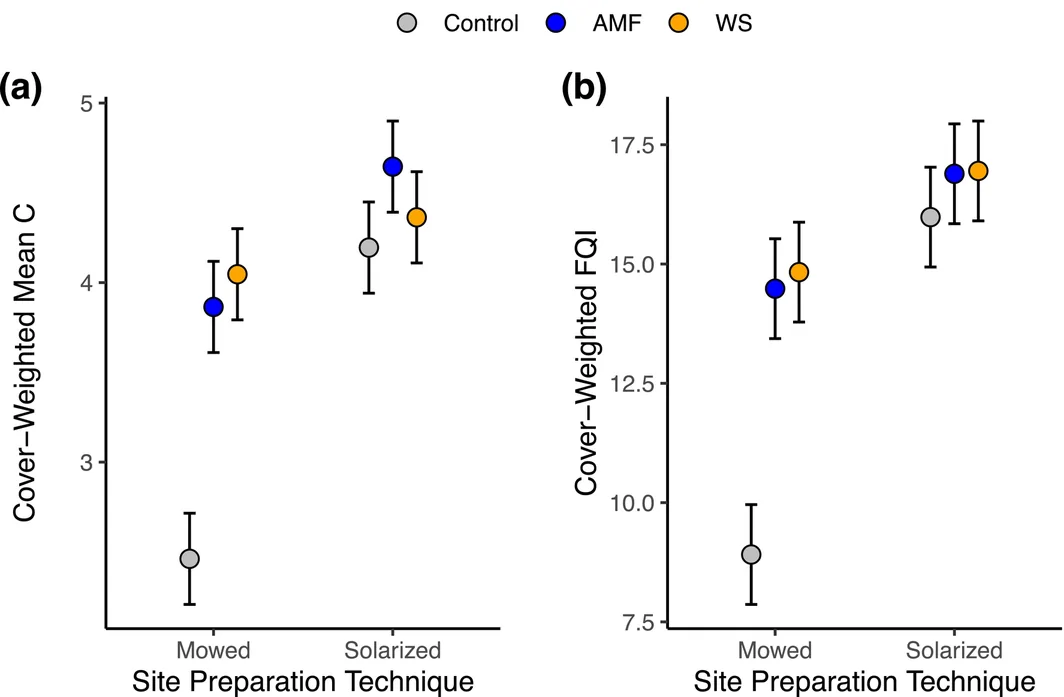
Mean (a) coefficient of conservatism (C) scores and (b) Floristic Quality Index (FQI) scores (both weighted) from plant communities that developed in field restoration plots by the seventh growing season (Summer 2022), Lawrence, KS, USA. Plots were inoculated with arbuscular mycorrhizal fungi (AMF), whole prairie soil (WS), or remained uninoculated (Control, gray). Plots were either initially solarized or initially mowed prior to planting.

Cover‐weighted CC scores were strongly affected by site preparation technique and microbial inoculation interactions (*F*_2,25_ = 4.04, *p* = 0.030; Figure 4a; Appendix S1: Table S6), with higher weighted CC scores in inoculated and solarized plots. Weighted Floristic Quality Index (FQI) scores were also impacted by site preparation technique and inoculation interactions (*F*_2,25_ = 3.53, *p* = 0.045; Figure 4b; Appendix S1: Table S6), with higher FQI in inoculated and solarized plots. Both mean weighed CC scores and mean weighted FQI scores were lowest in the uninoculated, mowed plots (Figure 4). Mean cover‐weighted CC and FQI scores for the second and fourth growing seasons are shown in Appendix S1: Figure S5, Appendix S1: Results S1.

In the seventh growing season, Shannon diversity was higher in the solarized plots (*F*_1,25_ = 13.1, *p* = 0.001; Appendix S1: Figure S6a). Native plant species richness was 1.3 times higher in the solarized plots (mean 12.5 ± 0.53) than in the mowed plots (mean 9.8 ± 0.53; *F*_1,25_ = 13.4, *p* = 0.001; Appendix S1: Figure S6b). However, there was no effect of inoculation on Shannon diversity (*F*_2,25_ = 0.332, *p* = 0.721; Appendix S1: Figure S6a) or native plant species richness (*F*_2,25_ = 0.27, *p* = 0.763; Appendix S1: Figure S6b), nor were there any site preparation by inoculation interactions on diversity (*F*_2,25_ = 1.84, *p* = 0.18; Appendix S1: Figure S6a) or native species richness (*F*_2,25_ = 2.69, *p* = 0.088; Appendix S1: Figure S6b). Native plant species richness and Shannon diversity for the second and fourth growing seasons are shown in Appendix S1: Figure S7, Appendix S1: Results S1.

#### Solarized plots and inoculated plots had less non‐native plant cover and greater late successional plant species cover

**FIGURE 5:**
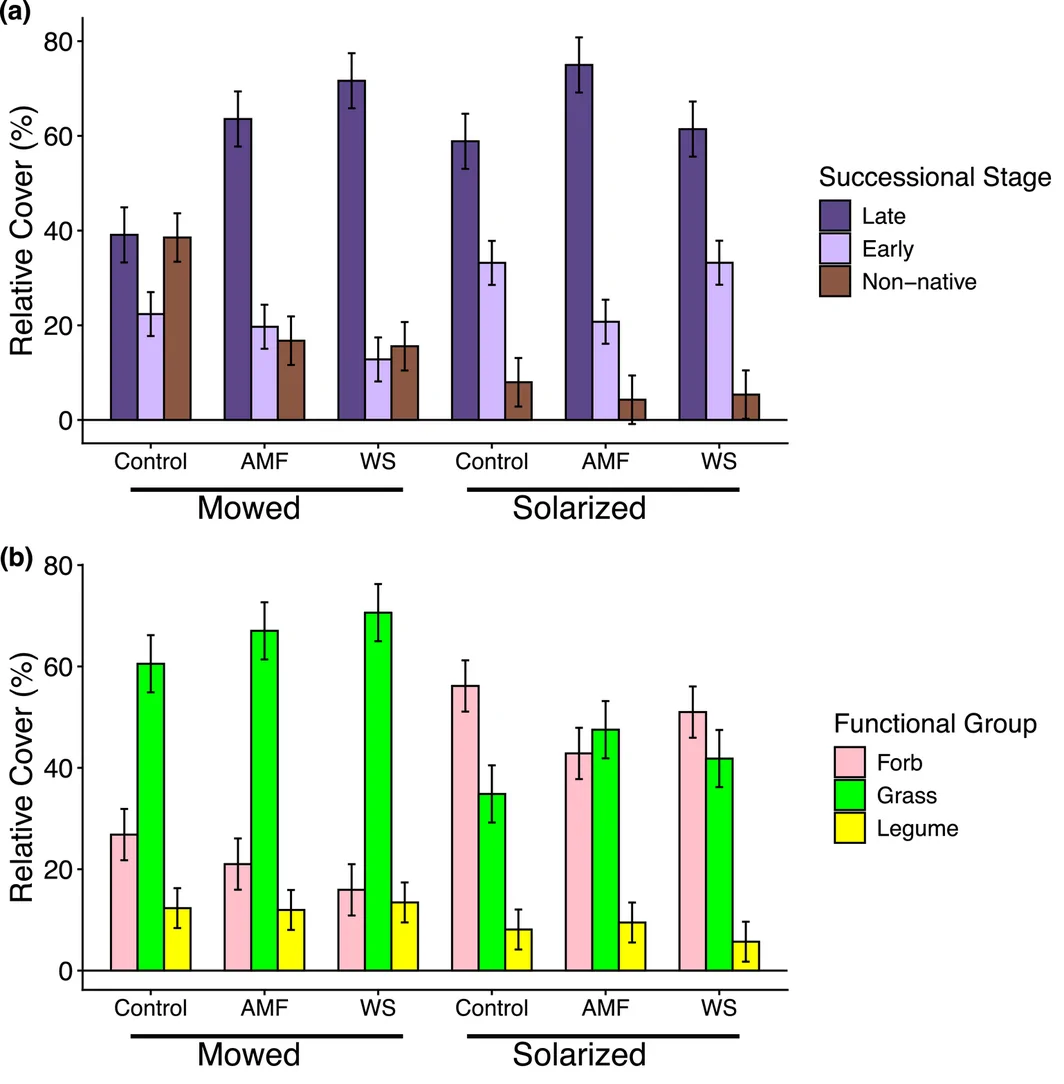
There were strong differences in the composition of the plant community that developed in the mowed plots and solarized plots. After seven growing seasons (a) relative cover of late successional (dark purple) and early successional (light purple) native plant species was affected by site preparation technique, microbial inoculation, and their interactions; relative cover of non‐native species (brown) was higher in mowed and uninoculated plots. After seven growing seasons (b) initially mowed plots were dominated by grasses (green) whereas relative cover of nonlegume forbs (pink) was higher in initially solarized plots. Legumes (yellow) remained in low abundance in both initially mowed and initially solarized plots. AMF, arbuscular mycorrhizal fungi; WS, whole prairie soil.

Initial solarization reduced non‐native plant cover by 17.8% compared to initially mowed plots (*F*_1,25_ = 18.0, *p* < 0.001; Figure 5a) and inoculation with AM fungi and whole prairie soil reduced non‐native plant cover by 12.7% and 12.8%, respectively, compared to uninoculated plots (*F*_1,25_ = 4.1, *p* = 0.028; Figure 5a). There were no site preparation techniques by inoculation interactions on non‐native plant cover (*F*_2,25_ = 2.38, *p* = 0.114), due to inoculation reducing non‐native biomass similarly across site preparation techniques. When the monoculture of the non‐native grass *B. inermis* was initially eliminated via solarization or mowed in May 2016 (Figure 1), it did not come back to dominate. In the seventh growing season, there was less than 1% relative cover of *B. inermis* in the solarized plots (mean 0% cover in the Control and AM fungal plots; mean 0.47% cover ±0.33% in the whole soil plots) and less than 25% relative cover of *B. inermis* in the mowed plots (mean 22.3% ± 9.7% relative cover in the Control plots; mean 10.4% ± 3.9% in the AM fungal plots; mean 8.2% ± 1.9% in the whole soil plots) (Appendix S1: Figure S8).

Site preparation technique, but not inoculation or their interaction, had strong effects on the relative percent cover of forbs (*F*_1,25_ = 48.4, *p* < 0.0001; Figure 5b) and grasses (*F*_1,25_ = 28.6, *p* < 0.001; Figure 5b). Throughout the study, initially mowed plots remained dominated by grasses (Figure 5b), whereas initially solarized plots contained a more even mixture of grasses and forbs (Figure 5b). Even after seven growing seasons and multiple reseedings, relatively few forbs (mean 21.3% ± 2.9% relative cover) established in the mowed plots compared to an average of 50.0% ± 2.9% relative cover of forbs in the solarized plots (Figure 5b). Late successional native plant species cover was higher in solarized and inoculated plots, as indicated by significant site preparation technique by inoculation interactions (*F*_2,25_ = 3.53, *p* = 0.045; Figure 5a). Early successional native plant species cover was higher in solarized compared to mowed plots (*F*_1,25_ = 8.1, *p* = 0.009; Figure 5a). The proportion of non‐native species in mowed and solarized plots in the second and fourth growing seasons (June 2017 and August 2019) is shown in Appendix S1: Figure S9; Appendix S1: Results S1, and relative cover of forbs, grasses, and legumes in the fourth growing season (August 2019) is shown in Appendix S1: Figure S10; Appendix S1: Results S1.

## DISCUSSION

Understanding the mechanisms that promote native seedling establishment in disturbed ecosystems is key to improving the biodiversity and function of landscape restorations. Here, we show that site preparation technique and soil microbial inoculations impart strong legacy effects on the development of plant communities in a grassland restoration. After seven growing seasons, field plots that were initially solarized to eliminate invasive *B. inermis* prior to restoration had a nearly 20% reduction in non‐native plant cover compared to plots that were initially mowed and overseeded. Plots that received native soil microbial inoculations had 13% lower non‐native plant cover compared to uninoculated plots, and non‐native abundance was reduced to near zero in inoculated and solarized plots. Abundance of late successional native plant species, the proportion of seeded native plants, mean weighted CC score, and mean weighted FQI were also higher in solarized and inoculated plots compared to plots that had been mowed or were uninoculated. Although native seedling establishment was higher overall in the solarized plots, the benefits of inoculation were greater in the mowed plots, in which native seedlings were planted directly into a monoculture of *B. inermis*. Our results support field studies showing that microbial inoculations can steer the outcomes of ecological restorations (Koziol, McKenna, et al., 2022; Koziol, Schultz, et al., 2022; Wubs et al., 2019), but show for the first time that microbial inoculations are beneficial whether or not invasive plant cover is removed prior to restoration efforts. Taken together, our results demonstrate that the way in which restoration sites are prepared, including whether or not microbial inoculations are used, can have strong and lasting impacts on the development of native plant communities. An increased abundance and diversity of desirable native plant species could enhance multifunctionality in ecological restorations by providing critical resources for a wide variety of organisms, including insects that rely on native plants for food and nesting resources, as well as providing vital ecosystem services, such as soil carbon storage, enhanced soil aggregate stability (Duchicela et al., 2012), reduced erosion (Grman et al., 2018), and reduced pathogen impacts (Wang et al., 2023).

### Inoculation improved nurse plant seedling survival and growth, with and without competition by *B. inermis*

Inoculation of nurse plant seedlings with native prairie microbes in a greenhouse prior to transplanting into the field increased the survival and growth of nurse plant seedlings in both the solarized and mowed plots. There was no difference in nurse plant survival or growth between seedlings inoculated with whole prairie soil or native AM fungi isolated and cultured from whole prairie soil, suggesting that the native AM fungi isolated from undisturbed (remnant) soils are key to promoting native plant establishment in grassland restorations. Our findings are consistent with recent tallgrass prairie restoration studies, in which inoculation with native prairie microbes, either AM fungi or whole prairie soil, also improved the establishment of native plant species across field sites (Koziol, Bauer, et al., 2022). Our study builds on past findings by demonstrating that the benefits of inoculation are stronger when native seedlings are in direct competition with an invasive species, compared to when they are planted into bare soil. Within the first year of the study, when native seedlings were transplanted into a monoculture of *B. inermis* in the mowed plots, over 70% of the uninoculated native seedlings died compared to 30% mortality of inoculated seedlings. Across both mowed and solarized plots, the C4 native bunchgrass, *S. scoparium* (little bluestem) had high survival and was a good competitor, regardless of inoculation status. Because *S. scoparium* established well in the restoration with and without competition by *B. inermis*, and is also a strong host plant for AM fungi (Cheeke et al., 2019), it makes a good nurse plant species to use for reintroducing native prairie microbes into tallgrass prairie restorations.

### Spread of benefits of inoculation to nearby seedlings

Although pre‐inoculating nurse plant seedlings with native microbes prior to transplanting into field plots clearly improved the survival and growth of the nurse plants themselves, our direct evidence that benefits spread to neighbors is limited. We designed a “runway” and “island” of uninoculated phytometers to explicitly test whether linkage of good host species at 0.5 meter spacing would facilitate AM fungal spread to 2 meters. However, we did not detect an effect over this distance. We note that amplicon sequencing identified that AM fungi can spread 2 m from points of inoculation (Tipton et al., 2022), and over this same distance, other inoculation studies detected benefits to uninoculated neighboring phytometers (Middleton et al., 2015; Middleton & Bever, 2012). Our power to detect effects was weakened by the high mortality of the uninoculated nurse plant and phytometer seedlings in the field. However, the poor survival of the uninoculated phytometers was consistent with the high mortality rates of the uninoculated nurse plants in the same plots, demonstrating the importance of native soil microbes to the survival of the mid‐to‐late successional native prairie plant species used in this experiment. In the future, planting with a greater diversity of native species with varying dependence on mycorrhizal fungi, or planting inoculated nurse plants at greater densities or in closer proximity to uninoculated seedlings, may better facilitate the spread of the microbes to neighbors and would be a fruitful area for future study. Indeed, a higher density of inocula was shown to be important in a field study (Koziol et al., 2020) and had increasing benefits over time likely due to positive feedbacks (Koziol, McKenna, et al., 2022; Koziol, McKenna, Duell, & Bever, 2025).

### Strong and lasting effects of site preparation technique on plant community composition

There were strong and lasting effects of site preparation technique on the development of plant communities, in which native plant species established better in solarized compared to mowed and overseeded plots. Throughout the course of the experiment and continuing into the seventh growing season, the mowed and overseeded plots consisted mostly of grasses, whereas the plots that had been solarized prior to planting contained a diverse mixture of forbs, grasses, and non‐forb legumes that successfully established from the seed mix. The higher abundance of forbs and lower abundance of non‐native grasses in the solarized versus the mowed plots was substantial, which could provide valuable floral resources to pollinators (Drobney et al., 2020).

The C3 grass *B. inermis* maintains its dominance in a landscape by creating dense sod that prevents other species from establishing (Mackiewicz‐Walec et al., 2024). In contrast to many native tallgrass prairie species of restoration interest, *B. inermis* shows little or no growth response to AM fungi (Cheeke et al., 2019), potentially providing them with a competitive advantage against later successional native grassland species in disturbed soils. Thus, restoring the belowground microbial community at the same time as aboveground restoration efforts (planting, seeding) may promote the growth of the native species of restoration interest while inhibiting the growth of their non‐native competitors.

### Inoculation with AM fungi, but not whole prairie soil, steered plant community composition in both solarized and mowed plots

After seven growing seasons, the plant communities in initially mowed and solarized plots were distinct when they contained nurse plants that had been inoculated with whole prairie soil or were uninoculated. However, in the plots that contained nurse plants that had been inoculated with AM fungi, the composition of the plant communities in initially mowed plots began to more closely resemble those in the initially solarized plots. This is due to the positive effect of inoculation on the abundance of highly desirable, late successional prairie plant species and suppression of weeds in mowed plots. Other studies have shown that inoculation can increase late successional prairie species (Koziol & Bever, 2017; Koziol, McKenna, Duell, & Bever, 2025) and suppress weeds (Koziol, McKenna, et al., 2022; Lubin et al., 2019). Although it is common for plant communities to change over time, this study shows distinct legacy effects of both the initial site preparation treatment (solarized vs. mowed) and the initial inoculation treatment (AM fungi, whole prairie soil, or uninoculated), even after seven growing seasons. To our knowledge, this is the longest inoculation experiment to date in which the effects of site preparation technique, with and without microbial inoculations, have been studied.

### Applications for future restorations

Overall, we found that the use of solarization, combined with microbial inoculations, successfully promoted the establishment of diverse native plant species in a restoration and that even when invasive ground cover was not eliminated prior to planting, the use of microbial inoculations also improved native species establishment. Because a diverse native plant community established from our seed mix particularly well in the solarized plots, our study demonstrates that tarping may be a good alternative to spraying or tilling for removal of invasive plants, especially for small‐scale restorations. Once *B. inermis* was eliminated via solarization, it did not come back, even after seven growing seasons, despite high propagule pressure from the near monoculture of *B. inermis* in the aisles and field edges. The use of tarps for solarization was also relatively low cost, required almost no maintenance besides periodic checking to make sure tarps were staked down, contained materials that could be reused for other restorations, and may have less impact on resident soil organisms compared to other management strategies. Other low‐cost mechanical barrier methods include applying cardboard after mowing, which blocks sunlight and suppresses invasive grasses (Gonçalves & Aximoff, 2021). However, for larger scale restorations, microbial inocula could be added to vast acreage via seed drills, adding to furrows, or hydroseeding (Koziol, 2017), while taking care to find a reputable inocula source (Jack et al., 2021; Koziol, McKenna, & Bever, 2025; Salomon et al., 2022). Patches of varying size and/or density of inoculated nurse seedlings could also be used as “nucleation” sites to provide habitat and serve as reservoirs for microbial dispersal into a restoration over time (Michaels et al., 2020). Although inoculation with remnant prairie soil can promote mycorrhizal colonization of native plant roots (Frewert et al., 2022) and improve native plant survival and growth (e.g., Cheeke et al., 2021; Koziol, Bauer, et al., 2022), reintroduction of native prairie soil can result in reduced benefits to native plants compared to reintroduction of native AM fungi alone (Reynolds et al., 2020), perhaps because of reintroduction of native pathogens. Moreover, harvesting soil from prairie remnants is not sustainable or practical for restoration at scale. However, our study shows that it is possible to use AM fungi isolated from prairie soil and bulk propagated in a laboratory as a sustainable and scalable method to increase late successional native plant establishment and reduce weeds while not requiring repeated harvesting from old‐growth donor sites.

More generally, our study adds to a compelling list of studies demonstrating that reintroduction of native AM fungi to tallgrass prairie restorations increases the success of desirable, late successional native plant species (Koziol et al., 2018). AM fungal benefits to plants are classically thought to be context dependent (Hoeksema et al., 2010). However, recent work has shown that the benefits of reintroduction of native AM fungi are consistent across land use histories (Koziol, Bauer, et al., 2022), across seeding density (Lubin et al., 2019), across levels of biochar addition (Frewert et al., 2022; House & Bever, 2020), across P and drought treatments (Bryant & Bever, 2024), in high‐nutrient soils (Cheeke et al., 2021), and even decades after initial prairie replanting (Bryant & Bever, 2025). While our study found that the benefit of native AM fungi is enhanced when prairie plants are competing with non‐native grasses, the study showed no downside to inoculation with solarization—a pretreatment that other studies have shown benefits (Koziol & Bever, 2017). This consistency of benefits across environmental contexts reflects the robust reality that high conservation value, late‐successional prairie plant species are very reliant on the late‐successional prairie AM fungi with which they have coevolved. It is likely that restoration of other similar “old‐growth” plant species in other systems will also depend upon reintroducing the particular symbionts on which they depend.

## AUTHOR CONTRIBUTIONS

Tanya E. Cheeke and James D. Bever conceptualized the research and designed the study. Tanya E. Cheeke set up and performed the experiment, collected data, conducted data analysis, interpreted the results, and acquired funding. Reb L. Bryant collected and analyzed plant composition data for 2022. Liz Koziol prepared the AM fungal inocula treatment, helped with plant identification and data collection, and provided feedback on the experimental design. Liz Koziol, James D. Bever, and Reb L. Bryant acquired additional funds to support data collection, data analysis, and/or writing. Gunner Davies participated in data collection. Tanya E. Cheeke wrote the first draft of the manuscript. Reb L. Bryant, James D. Bever, and Liz Koziol reviewed and edited the manuscript. All authors have read and agreed to the final draft of the manuscript.

## CONFLICT OF INTEREST STATEMENT

LK is the owner of MycoBloom LLC. MycoBloom did not participate in this study.

## Supporting information


Appendix S1.

## Data Availability

Data (Cheeke et al., 2026) are available in Dryad at https://doi.org/10.5061/dryad.fqz612k6s.
