# Supplementary material for: Inoculation with old‐growth microbes improves restoration quality and reduces non‐native encroachment after seven years
Source: Ecol Appl. 2026 Sep 8;36(6):e70295. doi: 10.1002/eap.70295 (PMC13553140; doi:10.1002/eap.70295)
Supplement: Supplementary file 1 [file EAP-36-e70295-s001.pdf]

Appendix S1

Inoculation with old-growth microbes improves restoration quality and reduces non-native encroachment after seven years

Tanya E. Cheeke, Reb L. Bryant, Gunner Davies, Liz Koziol, and James D. Bever

Ecological Applications

Table S1. Initial soil test results before soil inoculation treatments were applied (sampled May 2016) to field plots (Lawrence, KS, USA). Composite samples (one per plot) were air-dried and analyzed at Kansas State University (Manhattan, KS, USA).

Block	Plot	Plot Prep	OM %	pH	P-B ppm	K ppm	NH_4-N	$N0_3$-N ppm
1	1	Mowed	6.88	5.89	14.1	280	20.8	2.53
1	2	Solarized	7.60	5.78	15.4	293	7.97	1.54
1	3	Mowed	7.94	5.87	10.6	277	9.07	1.77
1	4	Solarized	7.14	5.90	13.8	335	7.02	12.39
1	5	Solarized	6.48	5.98	11.6	315	7.2	5.22
1	6	Mowed	6.55	6.17	8.95	286	13.2	4.09
2	7	Solarized	7.11	6.00	20.3	289	6.85	4.75
2	8	Solarized	7.62	6.06	16.9	306	8.65	10.61
2	9	Mowed	7.48	6.16	8.61	296	8.37	2.01
2	10	Mowed	7.48	6.35	8.05	307	6.51	0.68
2	11	Mowed	6.57	6.18	7.64	300	6.62	0.86
2	12	Solarized	6.83	5.90	13.2	324	7.72	10.82
3	13	Solarized	6.67	6.12	17.5	270	8.1	16.32
3	14	Mowed	6.03	6.02	7.92	237	6.95	1.79
3	15	Solarized	6.07	5.96	12.4	296	6.55	13.85
3	16	Solarized	6.67	6.17	12.5	309	8.35	8.85
3	17	Mowed	6.50	6.69	7.11	309	6.32	1.81
3	18	Mowed	5.66	5.99	5.07	248	6.6	2.52
4	19	Mowed	7.56	6.28	10.7	318	8.16	0.78
4	20	Solarized	7.48	5.84	16.8	325	8.99	11.61
4	21	Solarized	7.43	6.10	18	351	10.06	7.3
4	22	Mowed	7.41	6.83	15.9	299	9.19	2.3
4	23	Solarized	7.05	7.19	22.7	393	7	4.46
4	24	Mowed	8.18	6.64	13	373	10.75	6.03
5	25	Solarized	7.07	6.01	15.6	325	7.67	3.46
5	26	Mowed	6.44	6.21	8.2	307	8.08	1.15
5	27	Mowed	7.31	6.09	8.94	321	8.6	1.83
5	28	Mowed	6.46	7.24	11.9	373	6.89	2.6
5	29	Solarized	6.22	7.12	25.4	380	16.11	11.81
5	30	Solarized	6.40	6.28	14.1	351	9.42	1.33
6	31	Mowed	4.22	6.37	4.27	225	5.53	0.61
6	32	Mowed	6.64	6.16	7.51	317	7.93	0.42
6	33	Solarized	6.55	5.82	14	381	9.2	11.74
6	34	Solarized	8.07	6.02	19.9	453	11.29	1.32
6	35	Mowed	6.82	6.09	11	363	8.59	0.89
6	36	Solarized	6.78	6.15	18.3	430	9.07	5.46

Table S2. Inoculation treatments. Arbuscular mycorrhizal (AM) fungal inocula contained a mixture of seven different morphotypes isolated from remnant tallgrass prairies in Kansas and Missouri, USA and cultured in a greenhouse at Indiana University (Bloomington, IN, USA) in an autoclaved 1:1 mixture of Rockefeller prairie soil:Indiana River sand. Whole soil inocula consisted of live prairie soil (Rockefeller prairie, Lawrence, KS, USA) mixed 1:1 with autoclaved Indiana River sand. Control inocula contained a 1:1 mix of autoclaved AM fungal inocula and sand/soil mix.

Isolate (Lab ID)	Collection location (USA)	Inoculant description*	Putative ID	Treatment
207-2 KON 2015	Konza prairie, Manhattan, KS	1.20 L in the 7-species mix	*Claroideoglomus clariodium*	AM fungal inocula
205-2 KON 2015	Konza prairie, Manhattan, KS	0.95 L in the 7-species mix	*Glomus geosporum*	AM fungal inocula
300-1 Acul morrei MO 2015	Jefferson City, Missouri	1.60 L in the 7-species mix	*Acaulospora morrowiae*	AM fungal inocula
223-1 Rock 1M Gig. Gigaspora, pot A	Rockefeller prairie, Lawrence, KS	1.35 L in the 7-species mix	*Gigaspora gigantea*	AM fungal inocula
224-1 2I Rock 2015, Koseki, pot A	Rockefeller prairie, Lawrence, KS	2.15 L in the 7-species mix	*Acaulospora koskei*	AM fungal inocula
305-1 MO scut. Dip 2015	Jefferson City, Missouri	1.35 L in the 7-species mix	*Scutellospora dipurpurascens*	AM fungal inocula
226-1 Gl. Moss Rock DOD 3e	Rockefeller prairie, Lawrence, KS	1.50 L in the 7-species mix	*Funneliformis mosseae*	AM fungal inocula
Whole prairie soil inocula	Rockefeller prairie, Lawrence, KS	Mixed 1:1 with autoclaved Indiana River sand	Mixture of local AM fungi	Whole soil inocula
302B	Welda prairie Welda, KS	Mixed 1:1 with background sand/soil mix	Autoclaved	Control

*Volume based on availability of each isolate

Table S3. Local ecotypes of native prairie species seeded and/or transplanted into each plot. The four native 'nurse plant' and phytometer species that were transplanted as seedlings ('plugs') into field plots are indicated in **bold**. Coefficient of Conservatism (CC) scores indicating early successional (0-4) and late successional (5-10) native plant species are from the Floristic Quality Assessments database, Missouri region, 2015 (https://universalfqa.org). Species seeded in 2016 were obtained from Missouri Wildflowers (Jefferson City, MO, USA) and species that were seeded in 2021 were obtained from different sources, as indicated in the table.

**Botanical Name**	**Common Name**	**CC Score**	**Observed in 2nd growing season (2017)**	**Observed in 4th growing season (2019)**	**Observed in 7th growing season (2022)**
**Seeded or planted in 2016**					
Andropogon gerardii	Big bluestem	5	Present	Present	Present
Agalinis spp. (formerly Gerardia spp.)	Slender false foxglove	unknown	Not observed	Not observed	Not observed
Asclepias incarnata	Swamp milkweed	4	Not observed	Not observed	Not observed
Asclepias syriaca	Common milkweed	0	Not observed	Present	Not observed
Asclepias tuberosa	**Butterfly milkweed**	**5**	Present	Present	Present
Asclepias verticillata	Whorled milkweed	2	Present	Present	Present
Baptisia alba	White wild indigo	6	Present	Present	Present
Bidens aristosa	Bur marigold	1	Present	Not observed	Present
Bouteloua curtipendula	Side oats grama	7	Present	Present	Not observed
Castilleja coccinea	Indian paintbrush	6	Not observed	Not observed	Not observed
Chamaecrista fasciculata	Partridge pea	2	Present	Present	Present
Coreopsis lanceolata	Lanceleaf coreopsis	5	Present	Present	Present
Coreopsis tinctoria	Plains coreopsis	1	Present	Present	Present
Dalea purpurea	Purple prairie clover	8	Not observed	Not observed	Not observed
Desmanthus illinoensis	Illinois bundleflower	3	Present	Present	Present
Echinacea pallida	**Pale-purple coneflower**	**7**	Present	Present	Present
Eryngium yuccifolium	Rattlesnake master	8	Not observed	Not observed	Not observed
Eurybia hemispherica (formerly Aster paludosus)	Southern prairie aster	9	Not observed	Not observed	Not observed
Grindelia lanceolata	Narrowleaf gumweed	3	Present	Present	Not observed
Lespedeza capitata	**Roundhead bush clover**	**6**	Present	Present	Present
Liatris pycnostachya	Prairie blazing star	6	Present	Present	Present
Lobelia spicata	Pale-spiked lobelia	5	Not observed	Not observed	Not observed
Monarda fistulosa	Wild bergamot	4	Present	Present	Present

Parthenium integrifolium	Wild quinine	6	Present	Present	Present
Penstemon digitalis	Foxglove beardtongue	3	Present	Present	Present
Pycnanthemum tenuifolium	Slender mountain mint	4	Not observed	Present	Present
Ratibida pinnata	Gray-headed coneflower	4	Present	Present	Present
Rudbeckia hirta	Black-eyed Susan	1	Present	Present	Present
Rudbeckia subtomentosa	Sweet coneflower	5	Present	Present	Present
Sabatia angularis	Rose gentian	4	Not observed	Not observed	Not observed
Schizachyrium scoparium	**Little bluestem**	**5**	Present	Present	Present
Senna marilandica	Wild senna	4	Not observed	Present	Present
Silphium integrifolium	Rosinweed	4	Present	Present	Present
Silphium terebinthinaceum	Prairie dock	5	Present	Present	Present
Solidago rigida	Rigid goldenrod	3	Present	Present	Present
Solidago ulmifolia	Elmleaf goldenrod	4	Not observed	Not observed	Not observed
Sorghastrum nutans	Indian grass	4	Present	Present	Present
Symphyotrichum novae-angliae	New England aster	4	Present	Present	Present
Verbesina helianthoides	Wing stem	4	Present	Present	Present
**Seeded in 2021**					
Allium canadense[1]	Wild garlic	2	NA	NA	Not observed
Amorpha canescens[2]	Leadplant	8	NA	NA	Not observed
Artemisia ludoviciana var. ludoviciana[3]	White sage	3	NA	NA	Present
Asclepias tuberosa subsp. interior[1]	Butterfly milkweed	5	NA	NA	Present
Asclepias viridis[3]	Green-flowered milkweed	5	NA	NA	Not observed
Astragalus canadensis[1]	Canadian milk vetch	6	NA	NA	Not observed
Dalea candida[2]	White prairie clover	8	NA	NA	Not observed
Desmodium canadense[2]	Showy tick trefoil	4	NA	NA	Present
Heuchera richardsonii[1]	Prairie alum root	6	NA	NA	Not observed
Koeleria macrantha[4]	June grass	6	NA	NA	Not observed
Liatris squarrosa[3]	Scaly blazing star	6	NA	NA	Not observed
Oenothera macrocarpa[3]	Missouri primrose	7	NA	NA	Not observed
Phlox pilosa[3]	Prairie phlox	6	NA	NA	Not observed
Solidago gigantea[1]	Late goldenrod	3	NA	NA	Not observed

Solidago speciosa[3]	Showy goldenrod	7	NA	NA	Present
Sporobolus heterolepis[1]	Prairie dropseed	6	NA	NA	Not observed
Tripsacum dactyloides[5]	Gama grass	5	NA	NA	Not observed
Zizia aurea[1]	Golden alexanders	5	NA	NA	Present

1 = Prairie Moon Nursery (Winona, MN, USA)
2 = Hamilton Native Outpost (Elk Creek, MO, USA)
3 = Missouri Wildflowers (Jefferson City, MO, USA)
4 = Stock Seed Farms (Murdock, NE, USA)
5 = Star Seed (Osborne, KS, USA)

Table S4. Seventh growing season (Summer 2022): PERMANOVA showing effects of site preparation technique, microbial inoculation, and their interactions on the plant community that developed in the restoration plots, sampled in Summer 2022 (Figure 3c), seven growing seasons after the initial seeding event in May of 2016.

**Effect**	**Df**	**SumOfSqs**	**R^2**	**F value**	**P value**
Site Prep	1	1.3201	0.20848	9.47	**0.001**
Inoculation	2	0.5393	0.08517	1.9343	**0.026**
Site Prep*Inoc	2	0.2905	0.04589	1.0421	0.388
Residual	30	4.1818	0.66046		

Table S5. Seventh growing season (Summer 2022): Effects of site preparation technique, microbial inoculation, and their interactions on the relative cover (%) of desirable plant species (i.e., those that were intentionally seeded or planted) in the restoration in Summer 2022.

			Desirable cover (% relative abundance)	
**Effect**	**Num DF**	**Den DF**	**F value**	**P value**
**Site prep**	1	25	8.24	**0.008**
**Inoculation**	2	25	7.67	**0.0025**
**Prep*Inoculation**	2	25	1.77	0.191
**Block**	5	25	1.39	0.260

Table S6. Seventh growing season (Summer 2022): Effects of site preparation technique, microbial inoculation, and their interactions on the mean weighted coefficient of conservation (C) scores and mean weighted Floral Quality Index (FQI) scores in field restoration plots in the seventh growing season (Summer 2022), Lawrence, KS, USA.

			**Mean C (weighted)**		**FQI (weighted)**	
**Effect**	**Num DF**	**Den DF**	**F value**	**P value**	**F value**	**P value**
**Block**	5	25	2.13	0.0949	6.896	**0.0004**
**Site Prep**	1	25	20.709	**0.0001**	20.486	**0.0001**
**Inoculation**	2	25	8.419	**0.0016**	6.819	**0.0043**
**Prep*Inoc**	2	25	4.039	**0.0302**	3.527	**0.0448**

Supplementary Figures

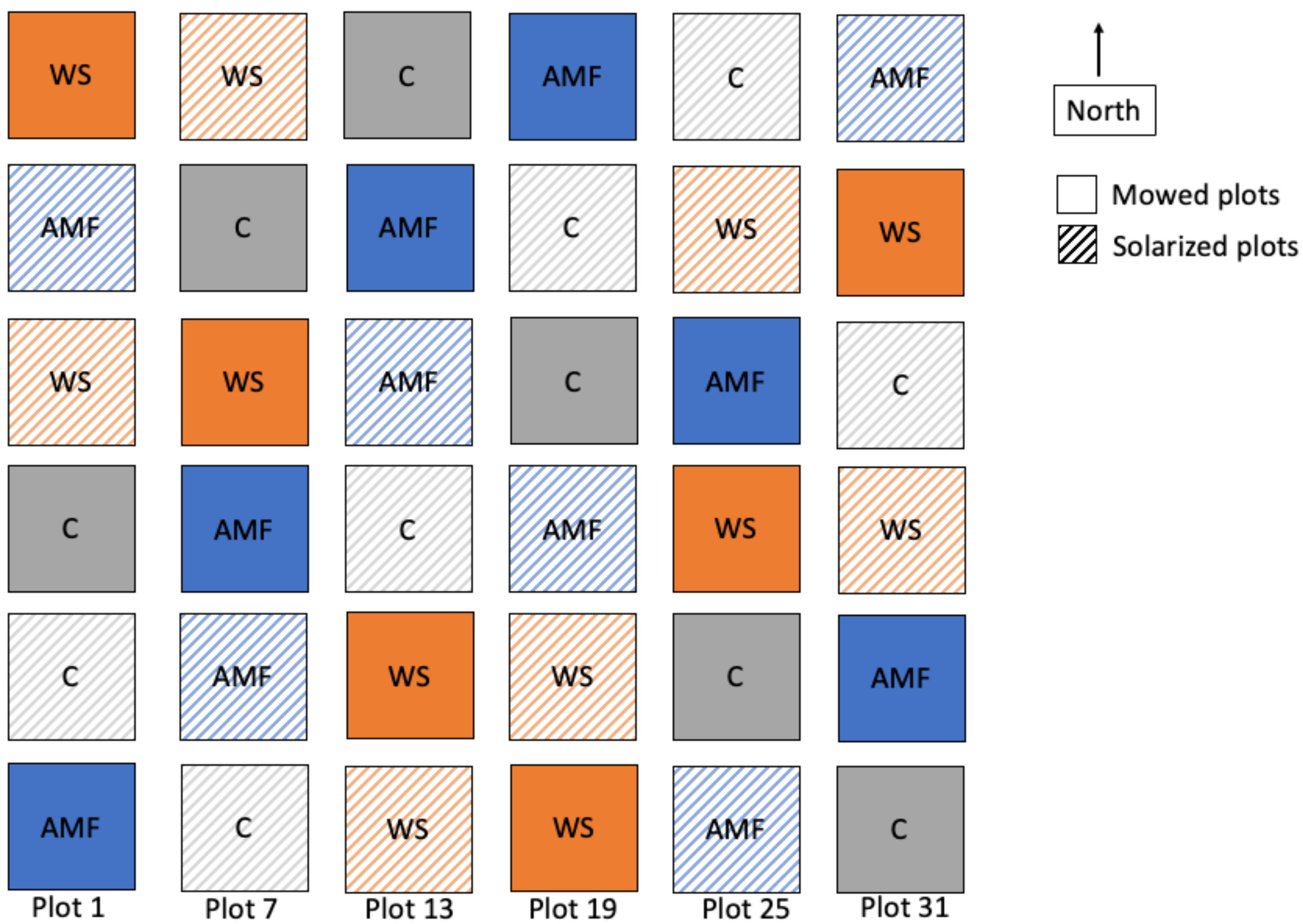

Figure S1. Field design: Latin square design showing field plots (5 x 5 m) of each treatment replicated exactly once in each row and column; 36 plots in total, with 3 m between plots and edges. Solid squares indicate plots that were mowed before planting; striped squares indicate plots that were solarized before planting. Plots in blue indicate the arbuscular mycorrhizal fungi (AMF) treatment; plots in orange indicate the whole prairie soil treatment (WS), and plots in gray indicate the uninoculated control treatment (C).

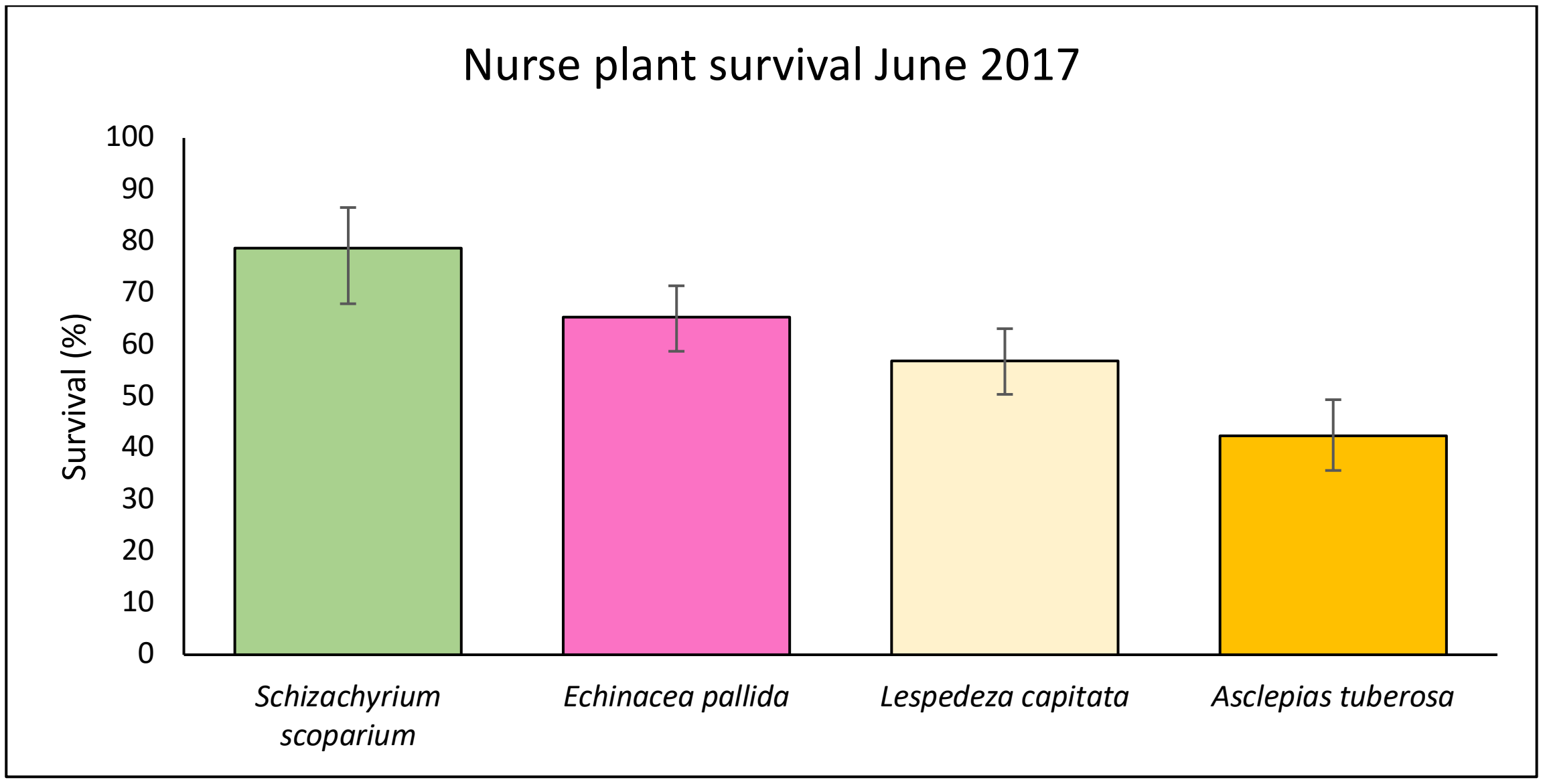

Figure S2. **Second growing season (June 2017):** Survival of native prairie seedlings *Schizachyrium scoparium, Echinacea pallida*, *Lespedeza capitata*, and *Asclepias tuberosa* in the second growing season. In June 2017, survival of the native grass *S. scoparium* was higher than other nurse plant species (P = 0.02, Table 1). Bars show the mean survival (%) +/- standard error.

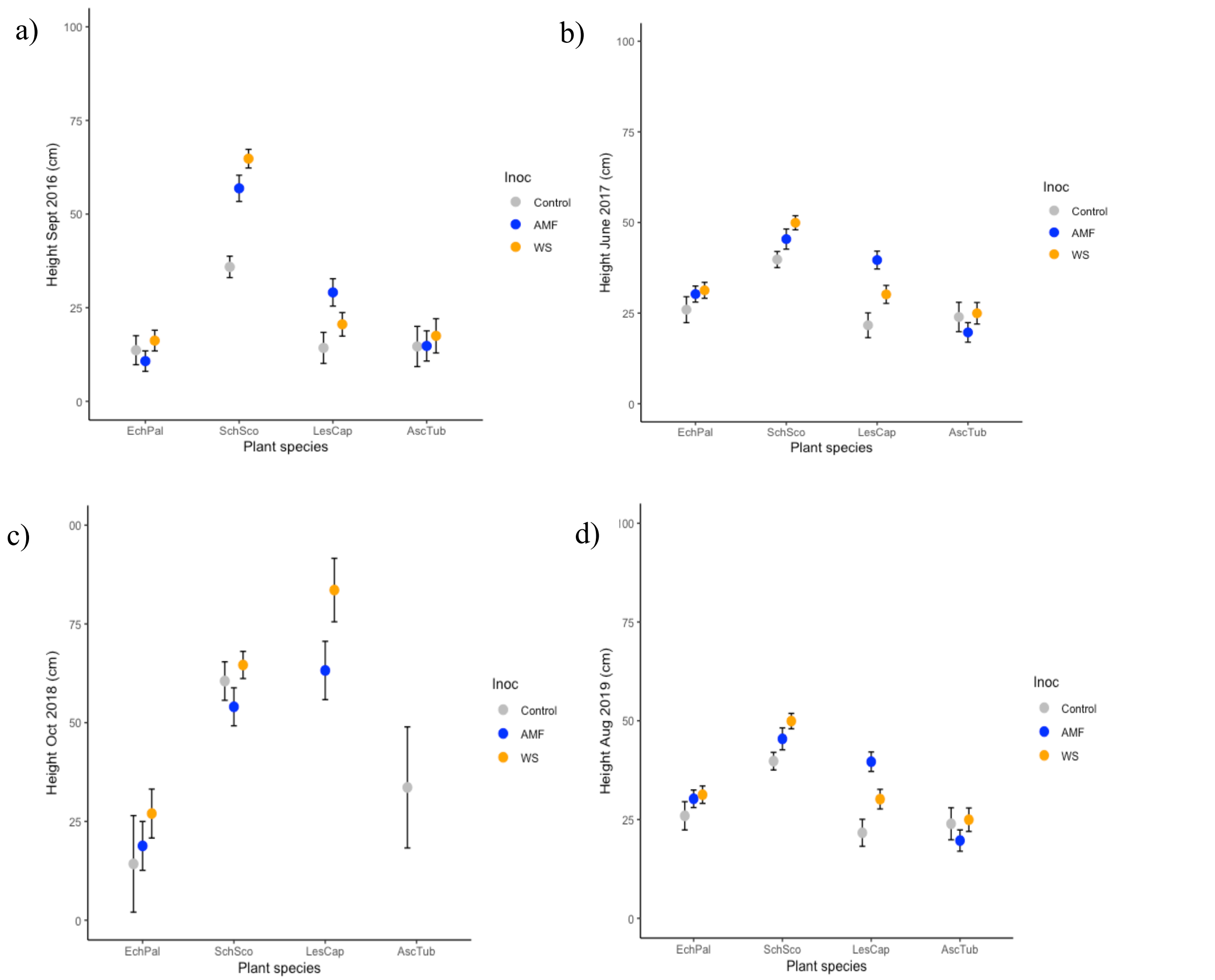

Figure S3. Nurse plant height from the first through the fourth growing season: Estimated marginal means and standard errors of nurse plant height (cm) in September 2016 (a, first growing season), June 2017 (b, second growing season), October 2018 (c, third growing season), and August 2019 (d, fourth growing season). Nurse plant species include *Echinacea pallida* (EchPal), *Schizachyrium scoparium* (SchSco), *Lespedeza capitata* (LesCap), and *Asclepias tuberosa* (AscTub), which were inoculated prior to planting into the restoration with native arbuscular mycorrhizal fungi (AMF, blue), whole prairie soil (WS, orange), or uninoculated (Control, gray). Species*Inoc interactions, $P < 0.04$; Table 2.

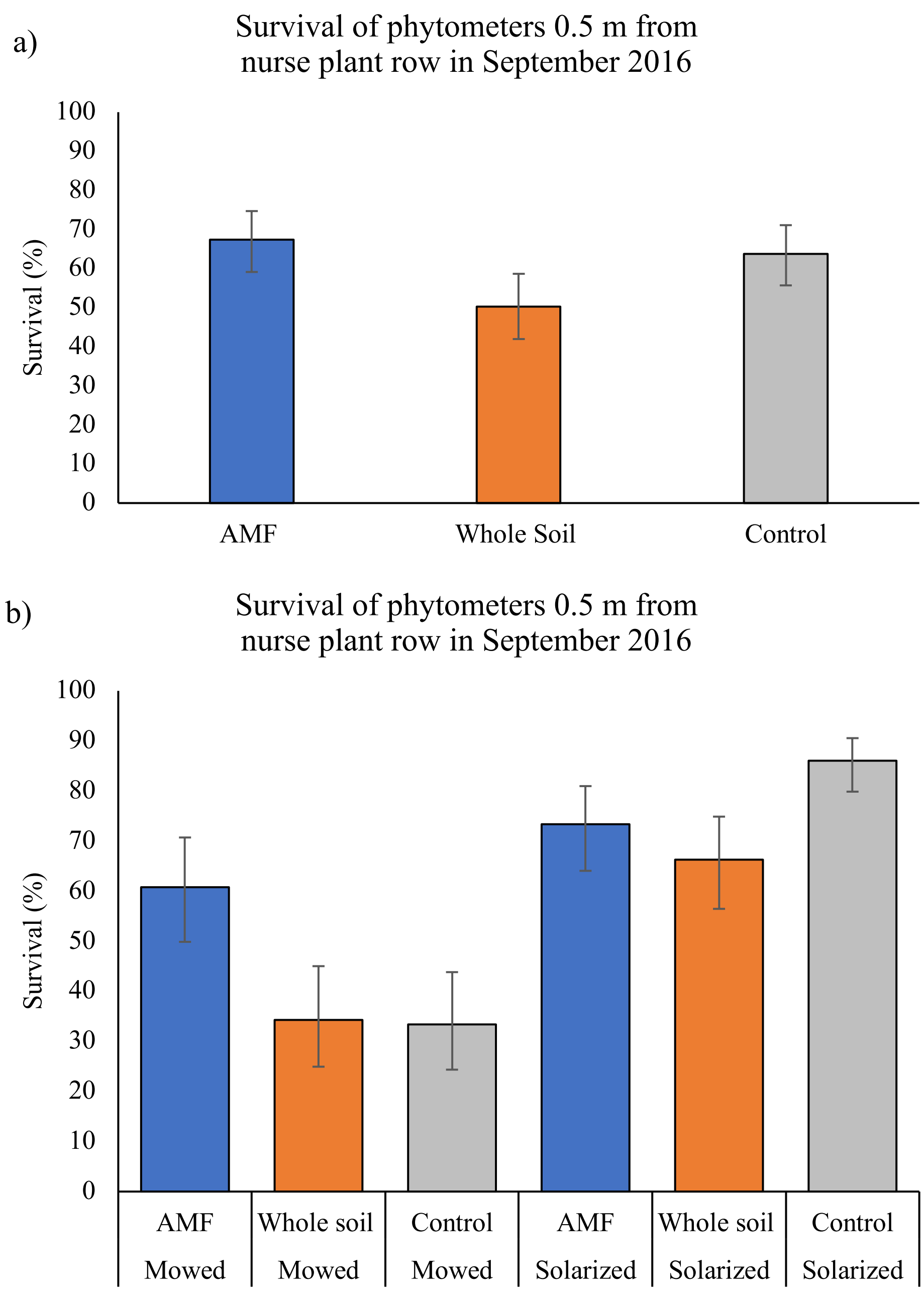

Figure S4. Survival of phytometers 0.5 m from nurse plant row four months after planting. Four months after transplanting (September 2016), phytometers that were planted 0.5 m from the nurse plant row had higher survival when grown (a) near nurse plants with the AM fungal inocula (AMF, blue) compared to those grown near nurse plants inoculated with whole prairie soil (orange), and (b) effects were stronger in the mowed compared to the solarized plots (solarization by inoculation effect, P = 0.0545; Table 3).

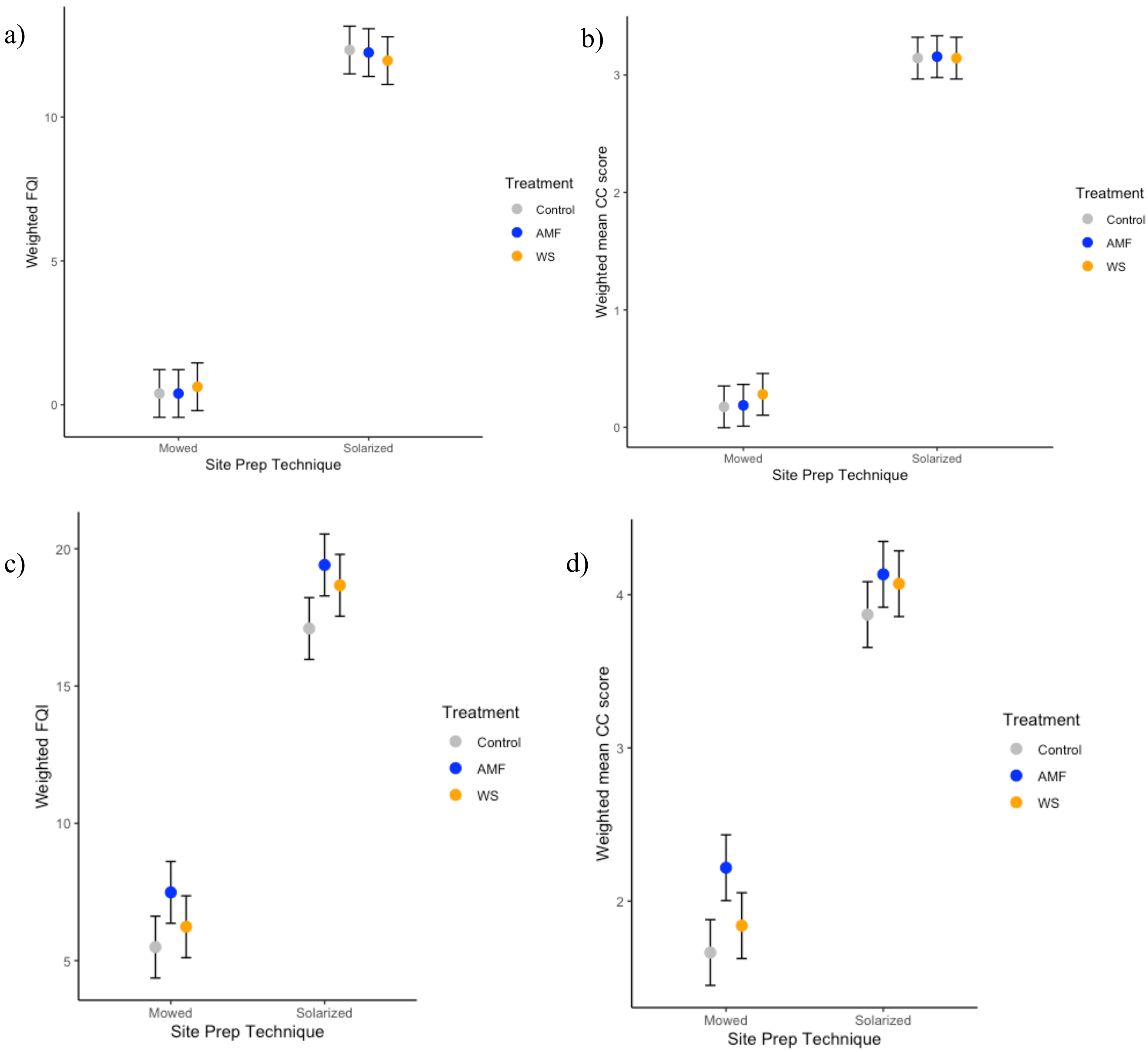

Figure S5. Weighted Floristic Quality Index scores and weighted mean Coefficient of Conservatism (CC) scores in June 2017 (a, b) and in August 2019 (c, d). In June 2017, (a) mean weighted FQI scores ($F_{1,29}$ = 298.95, $P < 0.0001$) and (b) mean weighted CC scores ($F_{1,29}$ = 408.6, $P < 0.0001$) were higher in solarized compared to mowed plots. In August 2019, (c) mean weighted FQI scores ($F_{1,29}$ = 169.97, $P < 0.0001$) and (d) mean weighted CC scores ($F_{1,29}$ = 146.05, $P < 0.0001$) were higher in solarized compared to mowed plots.

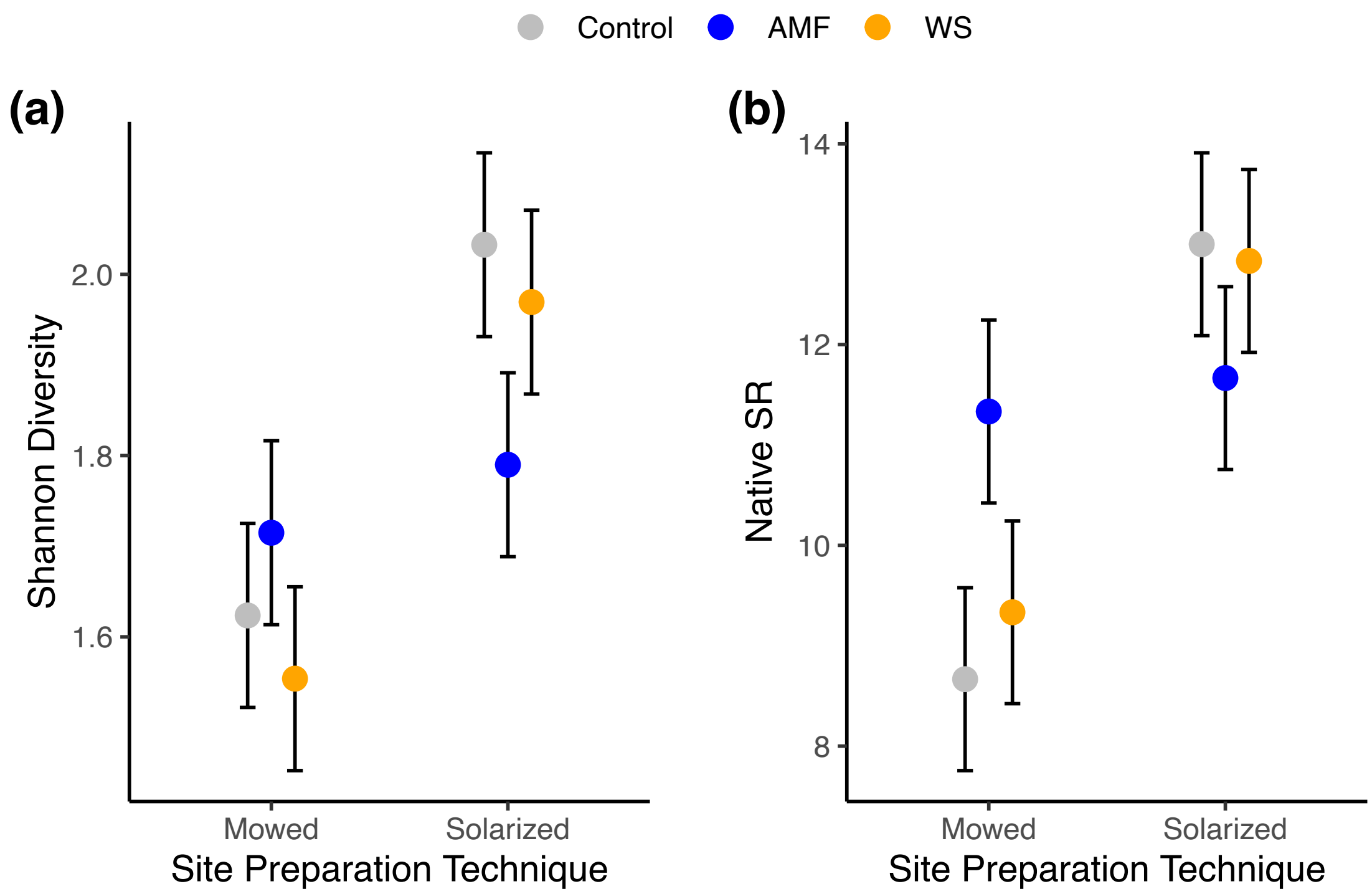

Figure S6. Seventh growing season (Summer 2022): (a) Shannon diversity was higher in solarized compared to mowed plots ($F_{1,25}$ = 13.10, P = 0.001), but there was no effect of inoculation ($F_{2,25}$= 0.33, P = 0.72) or site preparation technique x inoculation interactions ($F_{2,25}$ = 1.84, P = 0.18). (b) Native species richness (SR) was higher in solarized compared to mowed plots ($F_{1,25}$ = 13.41, P = 0.001), but there was no effect of inoculation ($F_{2,25}$= 0.27, P = 0.76) or site preparation technique x inoculation interactions ($F_{2,25}$ = 2.89, P = 0.09).

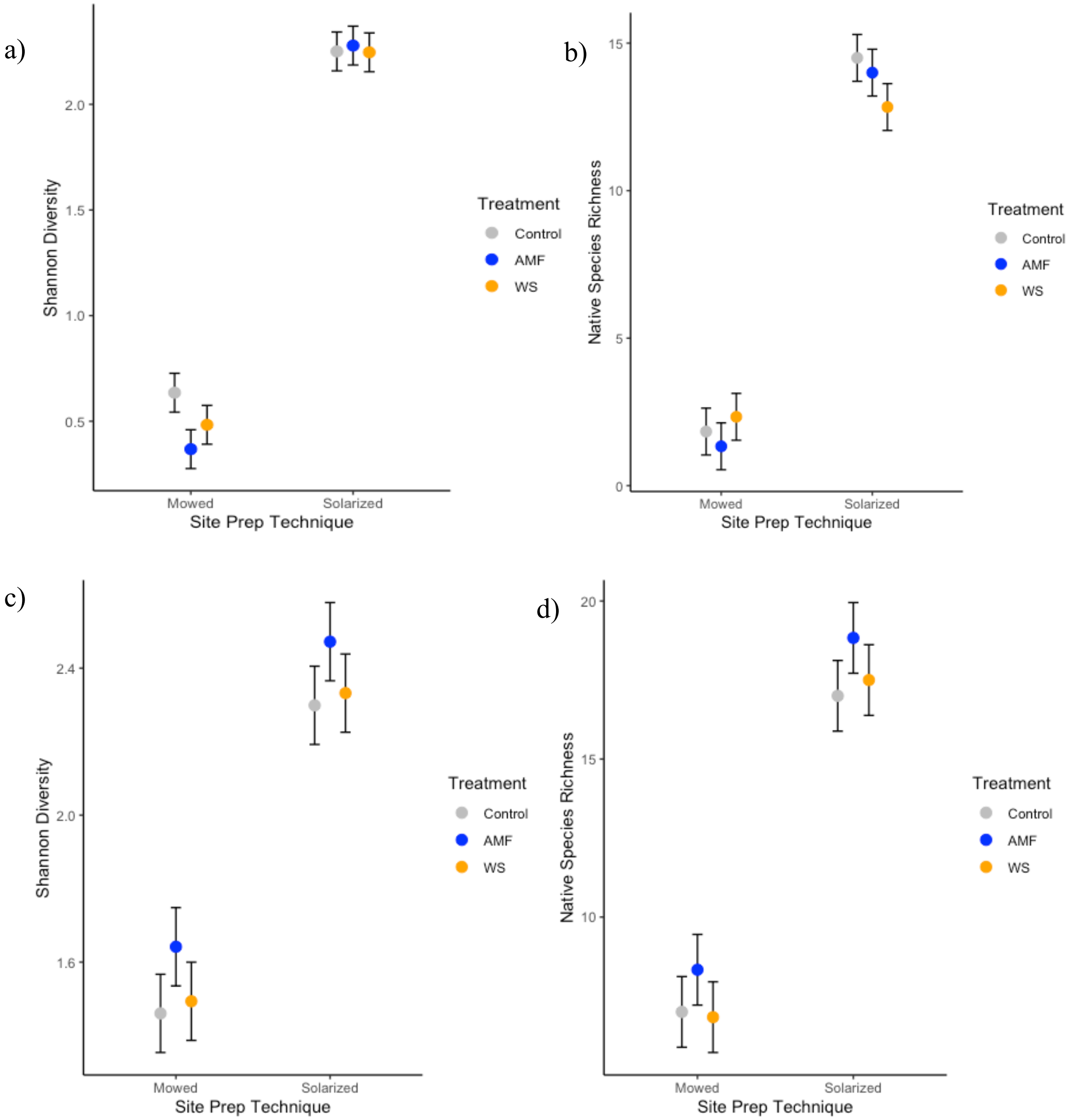

Figure S7. Shannon Diversity and Native Species Richness in June 2017 and August 2019: (a) Shannon diversity ($F_{1,29} = 551.23$, $P < 0.0001$) and (b) Native plant species richness ($F_{1,29} = 339.92$, $P < 0.0001$) were higher in solarized plots vs mowed plots in June 2017. In August 2019, Shannon diversity ($F_{1,29} = 92.47$, $P < 0.0001$) and Native plant species richness ($F_{1,29} = 129.43$, $P < 0.0001$) were also higher in the solarized vs mowed plots.

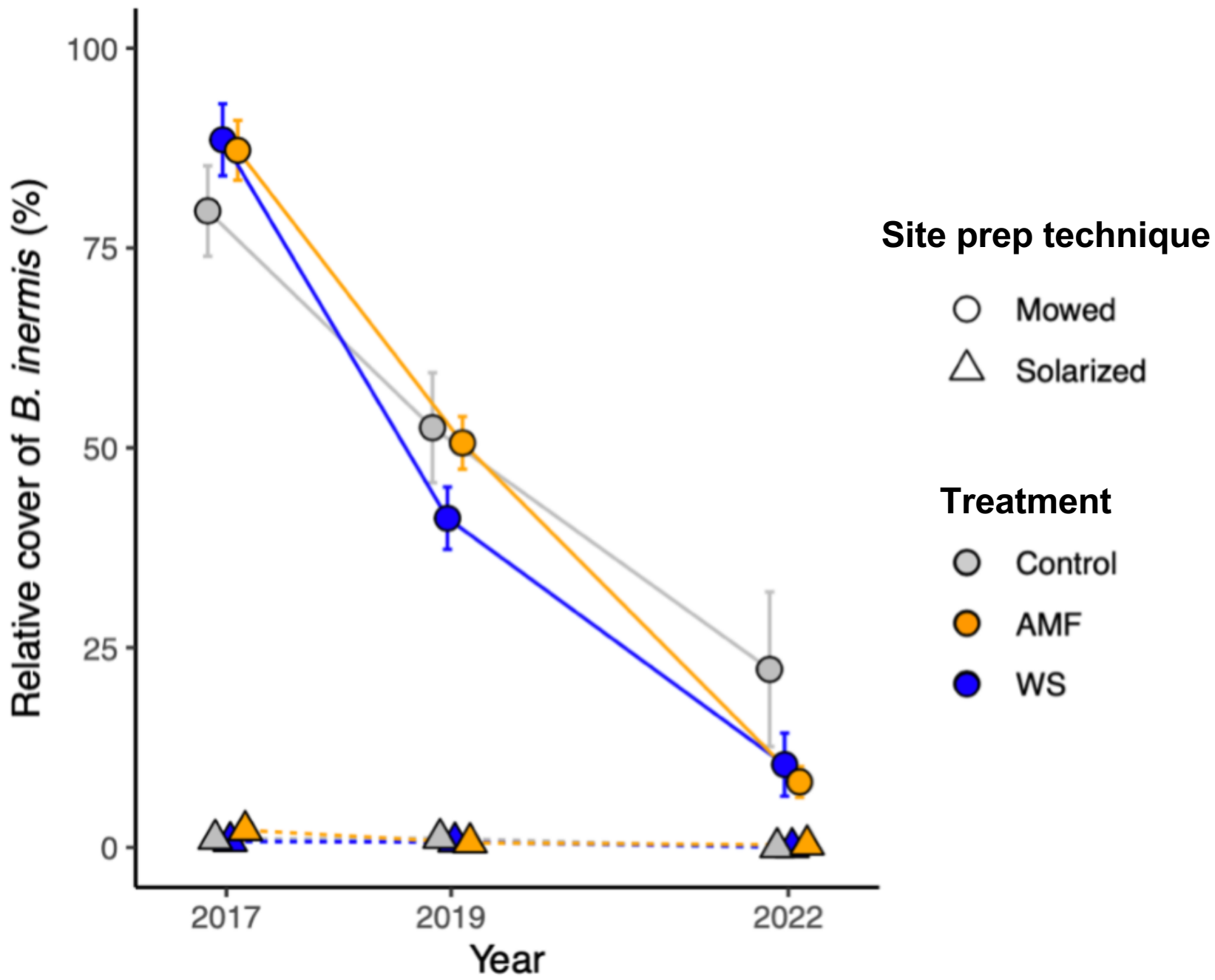

Figure S8. Relative cover of the invasive grass *Bromus inermis* in field plots that had been initially mowed (circles) or initially solarized (triangles). Plots were either initially mowed and overseeded or initially solarized and seeded in May 2016. In the seventh growing season (August 2022), there was less than 1% relative cover of *B. inermis* in the initially solarized plots (mean 0% cover in the Control and AM fungal plots, gray and blue triangles; mean 0.47% cover ± 0.33% in the whole soil plots, orange triangles) and less than 25% relative cover of *B. inermis* in the initially mowed plots (mean 22.3% ± 9.7% relative cover in the Control plots (gray circles), mean 10.4% ± 3.9% in the AM fungal plots (blue circles), and mean 8.2% ± 1.9% in the whole soil plots (orange circles).

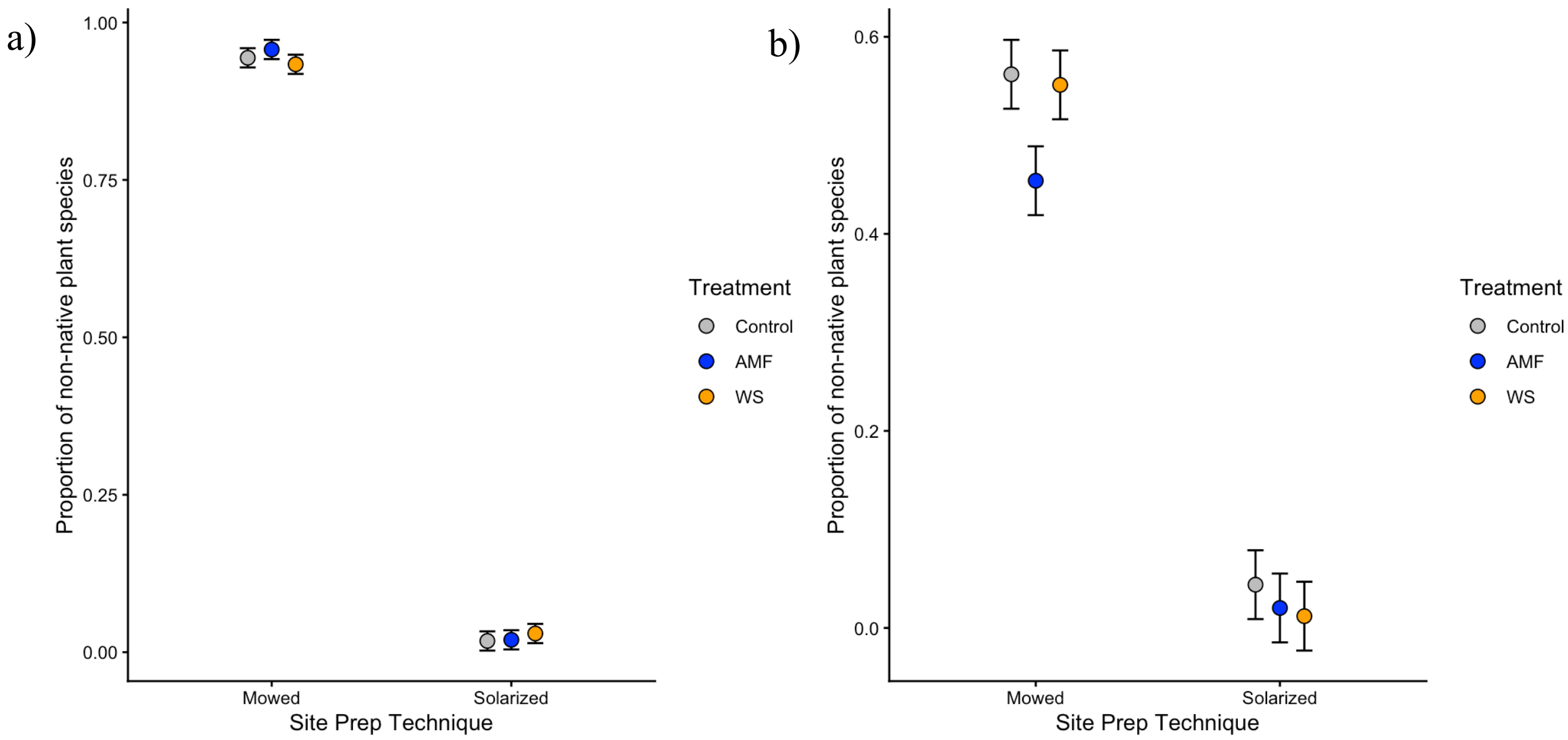

Figure S9. Proportion of non-native plant species in plots in June 2017 and August 2019. (a) In June 2017, mowed plots had a higher proportion of non-native plant species than solarized plots ($F_{1,25} = 8206.38$, $P < 0.0001$). (b) In August 2019, mowed plots had a higher proportion of non-native plant species than solarized plots ($F_{1,25} = 245.81$, $P < 0.0001$).

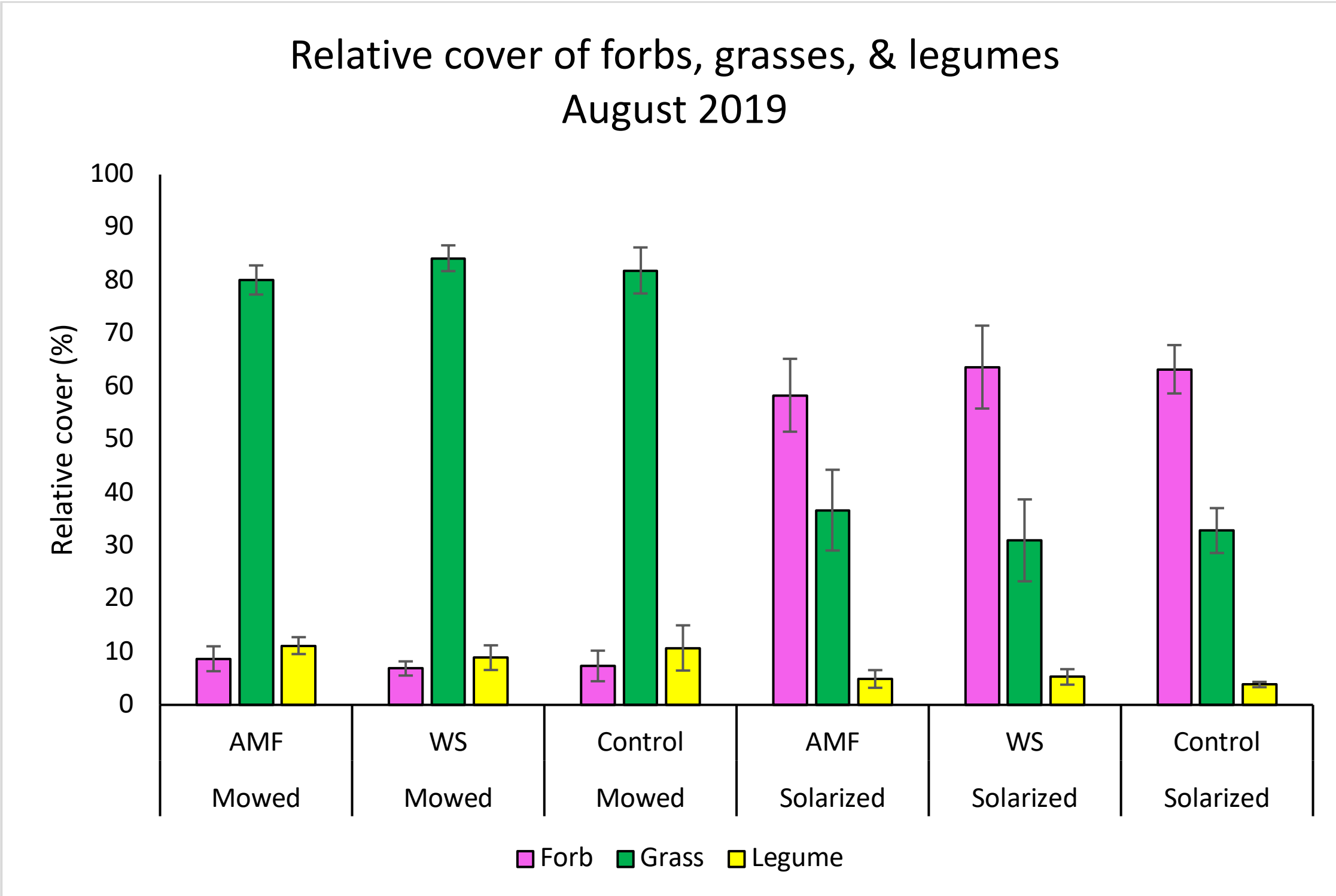

Figure S10. Fourth growing season (August 2019): In the fourth growing season, there was a strong effect of site preparation technique on the relative cover of non-legume forbs (pink), grasses (green), and legumes (yellow), but no effect of inoculation treatment was detected. Mowed plots remained mostly grasses (82%) and the relative cover of non-legume forbs was higher in solarized (62%) compared to mowed plots (8%). In the mowed plots, the non-native grass *Bromus inermis* made up 48% of the plant cover whereas it made up of < 1% of plant cover in solarized plots.

Results S1. Summary of plant community results from the second growing season (June 2017) and the fourth growing season (August 2019) in the restoration experiment.

Data collection: Plant community data reported for June 2017 were collected using the point line intercept method summed across five transects in each plot (2.0 m and 0.5 m on either side of the center row and the center row; Figure 1). Plant community data reported for August 2019 were collected from one quadrant of each plot, randomized across blocks. Different collection techniques were used depending on the stratification of the vegetation layers and density and diversity of the plant community as it developed over time, as well as availability of personnel. Different methods were cross-checked in the field at the beginning of each collection time to verify similar data collected across personnel and methods.

June 2017 (second growing season): In June 2017, 69% of the native species that had been originally seeded or planted in May 2016 were detected in the field plots. A total of 27 of the 39 seeded plant species had established, which included 13 late successional and 14 early successional native plant species (Table S3). Mean weighted FQI scores in the second growing season were higher in the initially solarized compared to the initially mowed plots ($F_{1,29}$ = 298.95, P < 0.0001), but there was no effect of inoculation ($F_{2,29}$= 0.003, P = 0.996) or site preparation technique x inoculation interactions on weighted FQI scores ($F_{2,29}$ = 0.08, P = 0.93; Figure S5a). Mean weighted CC scores in the second growing season were higher in solarized compared to mowed plots ($F_{1,29}$ = 408.6, P < 0.0001), but there was no effect of inoculation ($F_{2,29}$= 0.05, P = 0.95) or site preparation technique x inoculation interactions on weighted CC scores ($F_{2,29}$ = 0.06, P = 0.94; Figure S5b). Native plant species richness and Shannon diversity were higher in the solarized vs mowed plots ($F_{1,29}$ = 339.92, P < 0.0001, $F_{1,29}$ = 551.23, P < 0.0001, respectively; Figure S7a,b). In June 2017, mowed plots had a higher proportion of non-native plant species than solarized plots ($F_{1,25}$ = 8206.38, P < 0.0001; Figure S9a) but there was no effect of inoculation ($F_{2,25}$ = 0.22, P = 0.80) or site preparation technique x inoculation interactions detected ($F_{2,25}$ = 0.93, P = 0.41) detected.

August 2019 (fourth growing season): In August 2019, 74% of the native species that had been originally seeded or planted in May 2016 were detected in the field plots. A total of 29 of the 39 seeded or planted species had established, including 12 late successional native plant species and 17 early successional native plant species (Table S3). Mean weighted FQI scores in the fourth growing season were higher in the initially solarized compared to the initially mowed plots ($F_{1,29}$ = 169.97, P < 0.0001; Figure S5c), but there was no effect of inoculation ($F_{2,29}$= 1.83, P = 0.18) or site preparation technique x inoculation interactions on weighted FQI scores ($F_{2,29}$ = 0.07, P = 0.93). Mean weighted CC scores in the fourth growing season were higher in solarized compared to mowed plots ($F_{1,29}$ = 146.05, P < 0.0001; Figure S5d), but there was no effect of inoculation ($F_{2,29}$= 1.812, P = 0.18) or site preparation technique x inoculation interactions on weighted CC scores ($F_{2,29}$ = 0.335, P = 0.72). Native plant species richness and Shannon diversity were higher in the solarized vs mowed plots ($F_{1,29}$ = 129.43, P < 0.0001, $F_{1,29}$ = 92.47, P < 0.0001, respectively; Figure S7c,d). In August 2019, *Bromus inermis* made up 48% of relative plant cover in the initially mowed plots and < 1% of relative plant cover in the initially solarized plots (Figure S8). In August 2019, mowed plots had a higher proportion of non-native plant species than solarized plots ($F_{1,25}$ = 245.81, P < 0.0001; Figure S9b) but there was no effect of inoculation ($F_{2,25}$ = 1.77, P = 0.19) or site preparation technique x inoculation interactions

detected ($F_{2,25}$ = 1.01, P = 0.38) detected. Solarized plots contained a diverse mixture of native forbs (62%) and grasses (33%), whereas the mowed plots remained dominated by grasses (82%) and had very few forbs (8%) (Figure S10).
